# Fluorinated MOF platform for selective removal and sensing of SO_2_ from flue gas and air

**DOI:** 10.1038/s41467-019-09157-2

**Published:** 2019-03-22

**Authors:** M. R. Tchalala, P. M. Bhatt, K. N. Chappanda, S. R. Tavares, K. Adil, Y. Belmabkhout, A. Shkurenko, A. Cadiau, N. Heymans, G. De Weireld, G. Maurin, K. N. Salama, M. Eddaoudi

**Affiliations:** 1 Functional Materials Design, Discovery and Development Research Group (FMD³), King Abdullah University of Science and Technology (KAUST), Thuwal, 23955-6900 Saudi Arabia; 2 Sensors Lab, Advanced Membranes and Porous Materials Center, Division of Physical Sciences and Engineering, King Abdullah University of Science and Technology (KAUST), Thuwal, 23955-6900 Saudi Arabia; 3 Institut Charles Gerhardt Montpellier (UMR CNRS 5253), Université Montpellier, Place Eugène Bataillon, 34095 Montpellier, Cedex 05 France; 4 Service de thermodynamique, Faculté Polytechnique de Mons, Université de Mons, 20 Place du Parc, B-7000 Mons, Belgium

## Abstract

Conventional SO_2_ scrubbing agents, namely calcium oxide and zeolites, are often used to remove SO_2_ using a strong or irreversible adsorption-based process. However, adsorbents capable of sensing and selectively capturing this toxic molecule in a reversible manner, with in-depth understanding of structure–property relationships, have been rarely explored. Here we report the selective removal and sensing of SO_2_ using recently unveiled fluorinated metal–organic frameworks (MOFs). Mixed gas adsorption experiments were performed at low concentrations ranging from 250 p.p.m. to 7% of SO_2_. Direct mixed gas column breakthrough and/or column desorption experiments revealed an unprecedented SO_2_ affinity for KAUST-7 (NbOFFIVE-1-Ni) and KAUST-8 (AlFFIVE-1-Ni) MOFs. Furthermore, MOF-coated quartz crystal microbalance transducers were used to develop sensors with the ability to detect SO_2_ at low concentrations ranging from 25 to 500 p.p.m.

## Abstract (web summary)

Removal of SO_2_ from flue gas is of prime importance to avoid its poisoning of CO_2_-seperating agents. Here, the authors demonstrate that two fluorinated metal–organic frameworks selectively remove SO_2_ from synthetic flue gas and can sense SO_2_ with p.p.m.-level detection using quartz crystal microbalance transducers.

## Introduction

Global warming and other environmental/ecological issues have forced our society to adopt stringent rules for industrial waste and be on the lookout for ways to improve indoor and outdoor air quality, including in industrial sites. One major form of industrial waste with an adverse effect on the environment is flue gas. Flue gas generated by large industries and power plants resulting from burning fossil fuel contains CO_2_ (at a low percent concentration), SO_2_ (500–2000 p.p.m.), NO_2_ (few p.p.m.), water vapor, and nitrogen (as the dominant gas)^1^. Although the concentration of SO_2_ in flue gas feed is low, it could be poisonous for most liquid- or/and solid-state-based CO_2_ separating agents. Therefore, the removal of SO_2_ from flue gas is of prime importance^2^. Current SO_2_ removal technology involves the irreversible acid–base reaction of SO_2_ with CaO to form CaSO_3_^3^_._ The main drawback of this technology is its relatively low removal efficiency (< 90%) associated with an almost impossible regeneration step due to its extremely energy-intensive cost. Therefore, cyclable physical sorption technology is perceived as an effective alternative approach. Hence, identification of an adsorbent that can efficiently capture SO_2_, particularly at low concentration (< 500 p.p.m.), is crucial.

When SO_2_ is not controlled and is emitted into the atmosphere, it has adverse effects on the environment, such as acid rain, and must be monitored^4,5^. Therefore, it is necessary to find efficient solutions to sense SO_2_ at p.p.m. level (above 25 p.p.m.) in both dry and humid conditions. Recently, there have been considerable efforts in developing SO_2_ sensing devices based on metal oxides (such as SnO_2_, WO_3_, and TiO_2_) due to their excellent sensitivity, selectivity, response time, and recovery time^6–10^. However, most of the semiconductor-based SO_2_ sensors were reported to require high temperatures (200–600 °C), leading to high levels of power consumption^11–15^. There is therefore a need for gas sensors that operate at room temperature (RT)^16–18^, which would be an important parameter and invaluable milestone for developing alternate materials suitable for detecting SO_2_.

Metal–organic frameworks (MOFs), one of the most recent classes of porous materials, have attracted immense interest due to their potential to address many enduring challenges pertaining to various key applications^19–21^ such as separation, storage, catalysis, and sensing^22–24^. Although many of these MOFs possess good-to-excellent chemical stability (which is a prerequisite for practical industrial applications)^25,26^, there are only a few examples of SO_2_ adsorption on MOFs, mainly due to its corrosive nature^27–36^. Many of these reported MOFs adsorb SO_2_ irreversibly or undergo phase transformations and they are not suitable for SO_2_–structural-adsorption-sensing relationships studies; only a handful of MOFs are proven for cyclic stability and SO_2_ capture at flue gas concentration^27–30^. In addition, designing a sensor device for reversible SO_2_ sensing at p.p.m. level and in the presence of atmospheric moisture is still an ongoing challenge.

Here we report the use of isostructural fluorinated MOFs for (i) selective removal of SO_2_ from synthetic flue gas and (ii) sensing of SO_2_^37,38^ using a quartz crystal microbalance (QCM) as a transducer^18,39–41^, as the coating of MOFs on the QCM electrodes can detect the change in mass of sub-nanograms upon adsorption or desorption of molecules by the MOF layer^42,43^. We unveiled an unprecedented concurrent removal of SO_2_/CO_2_ from synthetic flue gas and remarkable detection capability in p.p.m. level of SO_2_ concentration in both dry and humid conditions.

## Results

### SO_2_ removal from flue gas

**Fig. 1:**
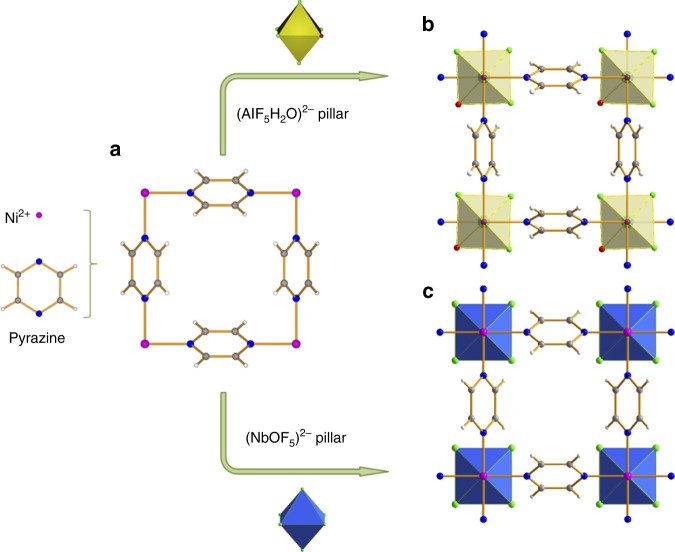
Synthesis and crystal structure of KAUST-7 and KAUST-8. **a** [Ni(pyrazine)_2_]^2+^ square grid resulting from the connection of Ni^2+^cations and pyrazine molecules. **b** Crystal structure of KAUST-8 resulting from the pillaring of [Ni(pyrazine)_2_]^2+^ square grid with (AlF_5_(OH_2_))^2−^ pillar. **c** Crystal structure of KAUST-7 resulting from the pillaring of [Ni(pyrazine)_2_]^2+^ square grid with (NbOF_5_)^2−^ pillar

Recently, our exploration of fluorinated MOF platforms, namely KAUST-7 ([Ni(NbOF_5_)(pyrazine)_2_]**·**2H_2_O, apparent surface area 280 m^2^ g^−1^, estimated pore volume 0.095 cm^3^ g^−1^) and KAUST-8 ([Ni(AlF_5_(OH_2_)) (pyrazine)_2_]**·**2H_2_O, apparent surface area 258 m^2^ g^−1^, estimated pore volume 0.102 cm^3^ g^−1^), resulted in many desirable properties that include direct air capture^44^, propane-propylene separation^45^, gas/vapor dehydration^46^, and acid gas (H_2_S, CO_2_) removal^47^. Although both of the MOFs are isostructural, the subtle differences in their chemical compositions, pillared by the inorganic moiety (NbOF_5_)^2−^ instead of (AlF_5_(OH_2_))^2−^, allowed the modulation of their properties by varying the content and intermolecular spacing of pending fluoride groups realized via different tilts of pyrazine molecules (Fig. 1). Inspired by the excellent stability and the modular nature of these MOF materials^44–46^, we investigated their SO_2_ removal and sensing in synthetic flue gas and air, respectively.

**Fig. 2:**
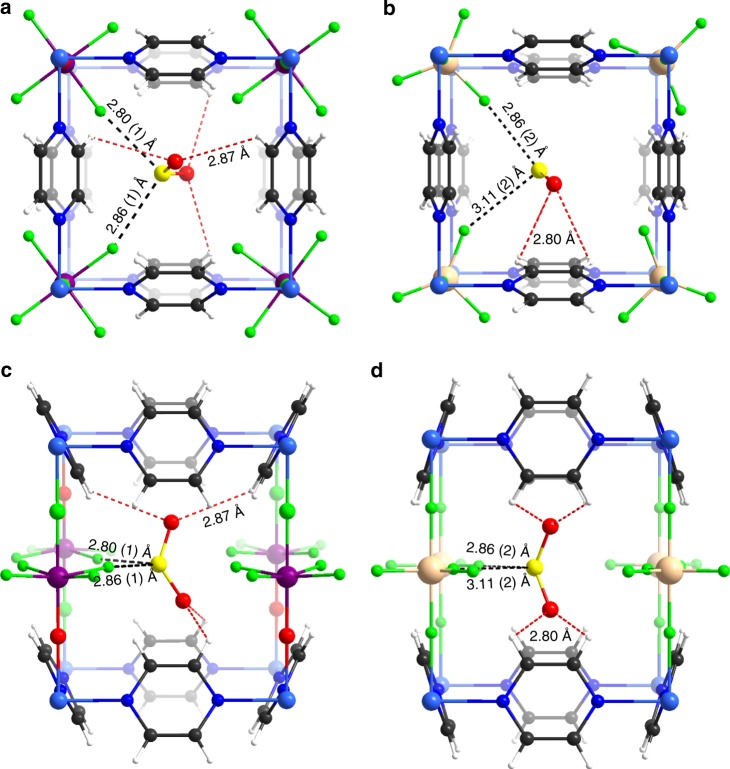
In situ visualization of SO_2_ in host frameworks. Intermolecular host–guest interactions in SO_2_ loaded crystal structures of **a**, **c** KAUST-7 and **b**, **d** KAUST-8

KAUST-7 was first investigated for SO_2_ sorption, which is also one of the best physisorbent materials for capturing CO_2_ at atmospheric concentration^44^ with good water stability^45^. The steep, pure SO_2_ adsorption isotherm collected at 25 °C (Supplementary Figure 1) suggested a high affinity of the KAUST-7 framework for SO_2_. To gain better insight on the SO_2_ position within the framework and the governing interactions responsible for resultant strong affinity, we performed in situ single crystal X-ray diffraction (SCXRD) study on a suitable single crystal of KAUST-7 at 296 K under 4–5 bar of SO_2_-containing atmosphere (7% SO_2_, 93% N_2_). The structure analysis revealed that the resultant compound crystallized in the tetragonal space group *P*4/*nbm* with the unit cell parameters *a* = *b* = 9.9249(2) Å and *c* = 7.8387(2) Å (Supplementary Table 1). An axial distortion of the octahedral (NbOF_5_)^2−^ anion (Nb–O bond is shorter than Nb–F_*trans*_, 1.82(3) and 2.03(3) Å, respectively) was observed and manifested in a disorder in the structure: the Nb atom was split over two positions, whereas the oxygen and the *trans*-fluoride were refined in the same position with equal thermal parameters (Supplementary Figure 2a). The fluorinated pillars are turned by *φ* = ±8.5(2)° from the direction toward the center of the channel (Supplementary Figure 2b) depending on the central atom position. The (NbOF_5_)^2−^ anion twist is stabilized by four pairs of C–H∙∙∙F hydrogen bonds with the adjacent pyrazine molecules: the H∙∙∙F distances and C–H∙∙∙F angles are 2.45 Å and 158° in the case of the Nb shifted toward that Ni(pyrazine)_2_ layer, and 2.52 Å and 170° when in the opposite direction (Supplementary Figure 2c and 2d). The SO_2_ molecule was localized in the center of the one-dimensional (1D) channel at a special position, similar to CO_2_ molecule in the KAUST-7 (CO_2_)structure^44^. As the site symmetry (−42*m*) is higher than the symmetry of the guest molecule, the SO_2_ is disordered over four positions around axis −4 with the total occupancy refined to 0.424(4). Contrary to the linear CO_2_ molecule surrounded by four F-pillars, the electropositive sulfur atom of the triangular SO_2_ molecule interacts with only two electronegative fluorine atoms of the adjacent (NbOF_5_)^2−^ anions (S∙∙∙F distances of 2.80(1) and 2.86(1) Å) shown in Fig. 2a–c. In addition, the SO_2_ molecule in the MOF is stabilized by four C–H∙∙∙O contacts with the hydrogen atoms of four different pyrazines (H∙∙∙O distance = 2.87 Å, angle = 120°).

This observation is corroborated by density functional theory (DFT) calculations, which revealed high SO_2_/KAUST-7 interaction energy of − 64.8 kJ mol^−1^. This is due to a relatively stronger interaction between the sulfur atom of SO_2_ and the F-pillars with characteristic interatomic distances of 2.9 Å (Supplementary Figure 3a) along with a charge transfer between the guest and this region of the MOF. Interestingly, the SO_2_/KAUST-7 interaction energy is as high as the value calculated for CO_2_ (−54.5 kJ mol^−1^). This latter molecule occupies slightly different sites than SO_2_, implying an interaction of the guest molecule with both the F-pillars and the pyrazine groups (Supplementary Figure 3b). The so-predicted energetics and spatial distributions for both molecules as single components suggest simultaneous capture of SO_2_ and CO_2_.

**Fig. 3:**
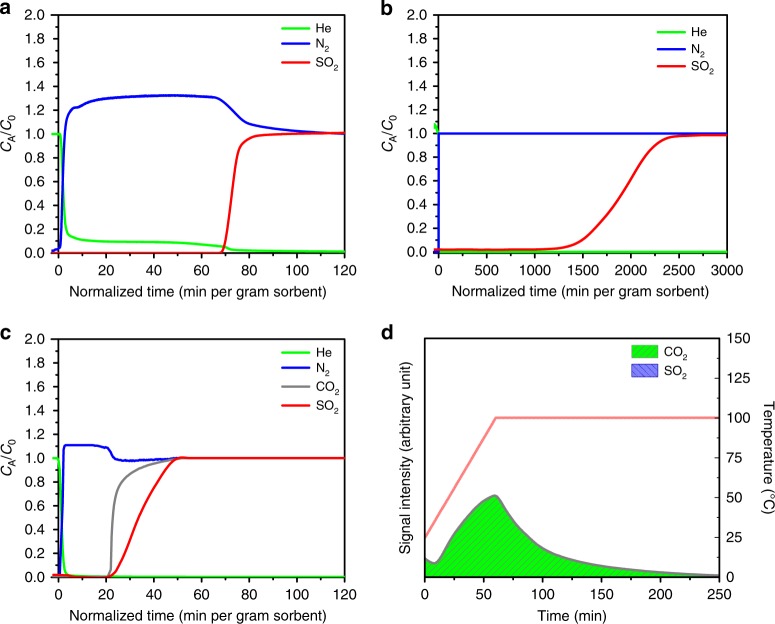
CO_2_/SO_2_ capture performance of KAUST**-7**. Adsorption column breakthrough experiments for KAUST**-7** with **a** SO_2_/N_2_: 7/93 mixture (10 cm^3^ min^−1^, flow rate), **b** SO_2_/N_2_: 0.05/99.95 mixture (40 cm^3^ min^−1^, flow rate), and **c** SO_2_/CO_2_/N_2_: 0.05/10/89.95 mixture (25 cm^3^ min^−1^, flow rate). **d** Temperature-programmed desorption after initial adsorption in the column using a mixture akin to flue gas (SO_2_/CO_2_/N_2_: 0.05/10/89.95), suggesting an adsorbed phase composition dominated by CO_2_

Cyclic adsorption column breakthrough tests with SO_2_/N_2_: 7/93 indicate stability and good uptake (≈2.2 mmol g^−1^) of SO_2_ (Fig. 3a). Furthermore, adsorption column breakthrough experiments with SO_2_/CO_2_/N_2_: 4/4/92 gas mixture showed simultaneous and equal retention time in the column for SO_2_ and CO_2_, demonstrating identical uptake of ≈1.1 mmol g^−1^ (Supplementary Figure 4), which is consistent with the simulated energetics trends. Upon decreasing the SO_2_ concentration with nitrogen in the range commonly observed in flue gas (500 p.p.m.) (SO_2_/N_2_: 0.05/99.95 mixture) and the immediately dangerous to life or health concentration (100 p.p.m.), KAUST-7 still maintains a high SO_2_ uptake of about 1.4 mmol g^−1^ at 500 p.p.m. SO_2_ concentration (Fig. 3b). Interestingly, adsorption column breakthrough experiments under mimicked flue gas conditions with 500 p.p.m. of SO_2_ and 10% CO_2_ in N_2_ (SO_2_/CO_2_/N_2_: 0.05/10/89.95) resulted in equal and simultaneous retention time for both SO_2_ and CO_2_, leading to uptakes of ≈0.01 mmol g^−1^ and ≈2.2 mmol g^−1^, respectively (Fig. 3c). The direct co-adsorption experiments with different SO_2_ and CO_2_ compositions (4% SO_2_, 4% CO_2_, balance N_2_ and 500 p.p.m. SO_2_, 10% CO_2_, balance N_2_) demonstrate that KAUST-7 exhibits equal selectivity toward SO_2_ and CO_2_ (SO_2_/CO_2_ selectivity ≈1), which is desirable for simultaneous CO_2_ and SO_2_ capture in flue gas (containing low SO_2_ concentrations). Nevertheless, temperature-programmed desorption (TPD) confirmed the presence of CO_2_ only with an undetectable amount of SO_2_ (Fig. 3d) in the adsorbed phase as the amount of SO_2_ adsorbed is negligible owing to its low concentration. The performance of KAUST**-7** using humid (≈40% Relative Humidity (RH)) 250 p.p.m. SO_2_/balance N_2_ gas mixture was also investigated (Supplementary Figure 5); it is evident that SO_2_ has same breakthrough time as that of water and results in a lower SO_2_ uptake under humid condition compared with the dry condition, which is not surprising considering the concentration of water is around 50 times higher than that of SO_2_. In addition to previously reported excellent water stability^44,45^, SO_2_ stability of the KAUST**-7** was proven by powder X-ray diffraction (PXRD) comparison of the materials before and after dry and humid SO_2_ exposure (Supplementary Figure 6), CO_2_ breakthrough study before and after 7% SO_2_ breakthrough experiments (Supplementary Figure 7), and CO_2_ adsorption isotherm after the humid SO_2_ breakthrough experiment (Supplementary Figure 8).

In our quest for a material with a more favorable selectivity for SO_2_ removal from flue gas than CO_2_ (at 500 p.p.m. of SO_2_), we opted to investigate an analog of KAUST**-7** with lower CO_2_ interactions and potentially higher SO_2_ interactions. Inspired by our results with KAUST**-**8 for dehydration of gases^46^ and simultaneous removal of H_2_S/CO_2_^47^, we found it compelling to explore the structural SO_2_/CO_2_ co-adsorption property. KAUST-8 exhibits three pendant fluoride groups with slightly higher F…F distance (3.613 Å) and one potential open metal site, whereas KAUST-7 contains four pendants fluoride with smaller F…F distance (3.210(8) Å) and no open metal site. Such minute differences in structural features led us to realize equal selectivity for CO_2_ and H_2_S over a wide range of concentrations and temperatures^47^. Encouraged by this structure–property tuning of H_2_S and CO_2_ adsorption affinity using this MOF, we expected KAUST-8 to be more selective toward SO_2_ than CO_2_. Similar to gain better insight of the KAUST-8 system, SCXRD data were collected on the SO_2_-loaded KAUST-8 crystal. The structure analysis revealed the resultant compound crystallized in the tetragonal space group *P*4/*mmm* with the unit cell parameters *a* = *b* = 6.9996(2) Å and *c* = 7.7033(2) Å (Fig. 2b–d, Supplementary Table 2). Contrary to KAUST-7, a rotational disorder of the (AlF_5_)^2−^ is caused by trigonal bipyramidal shape of the anions located at the axis 4 among four 1D channels in the structure. When one fluorine atom of a pillar is directed perfectly to the channel center (*φ* = 0°), in the two other adjacent channels Al–F bonds of the same anion are directed ± 24.1(4)° out the diagonal direction, and in the fourth channel there are no F-atoms from this pillar (Supplementary Figure 9a). Therefore, the single average cage aperture is formed by fluorine atoms of three adjacent (AlF_5_)^2−^ anions only. The pillar twist is stabilized by three pairs of C–H∙∙∙F hydrogen bonds between the pyrazine molecules and the fluorine atoms with H∙∙∙F distances and C–H∙∙∙F angles of 2.38 Å and 178°, 2.48 Å and 134°, and 2.17 Å and 161°, respectively (Supplementary Figure 9b and 9c). Similar to SO_2_-loaded KAUST-7 structure, the guest SO_2_ molecule is disordered in the center of the 1D channel (the special position symmetry is 4/*mmm*) of the KAUST-8 MOF. The distances between electropositive sulfur atom of the SO_2_ molecule and fluorine atoms of adjacent F-pillars equals 2.86(2), 3.11(2), or 3.25(1) Å depending on the (AlF_5_)^2−^ orientation (*φ* = 0° or ± 24.1(4)°, Supplementary Figure 9d) and each of them is less than the sum of the S and F van der Waals radii (3.27 Å). Contrary to the KAUST-7 structure, the SO_2_ molecule participates in four C–H∙∙∙O contacts with only two neighboring pyrazines (H∙∙∙O distance = 2.80 Å, angle = 105°). These observations were supported by DFT calculations and it revealed a lowering of the host/guest interaction energy of CO_2_ for KAUST-8 compared with KAUST-7 (− 47.0 kJ mol^−1^ vs. − 54.5 kJ mol^−1^). In the case of KAUST-8, the trigonal bipyramidal-like Al^3+^ environment does not allow for further optimal interactions between a carbon atom in CO_2_ and four F-pillars (Supplementary figure 3d), as seen in KAUST-7. DFT calculations were further performed starting with the SO_2_-loaded crystal structure model elucidated from SCD data. Interestingly, the simulated preferential location of SO_2_ is slightly pushed toward the pore wall, as compared with the scenario in KAUST-7, with the formation of a dual interaction between its sulfur atoms and the two nearby F-pillars, as well as its oxygen atoms interacting with the pyrazine linker with shorter interacting distances (Supplementary Figure 3c). The resulting geometry led to a slight enhancement of the SO_2_/host interaction energy (− 73.9 kJ mol^−1^) and reduced affinity toward CO_2_, making KAUST-8 a promising candidate to selectively adsorb SO_2_ over CO_2_.

**Fig. 4:**
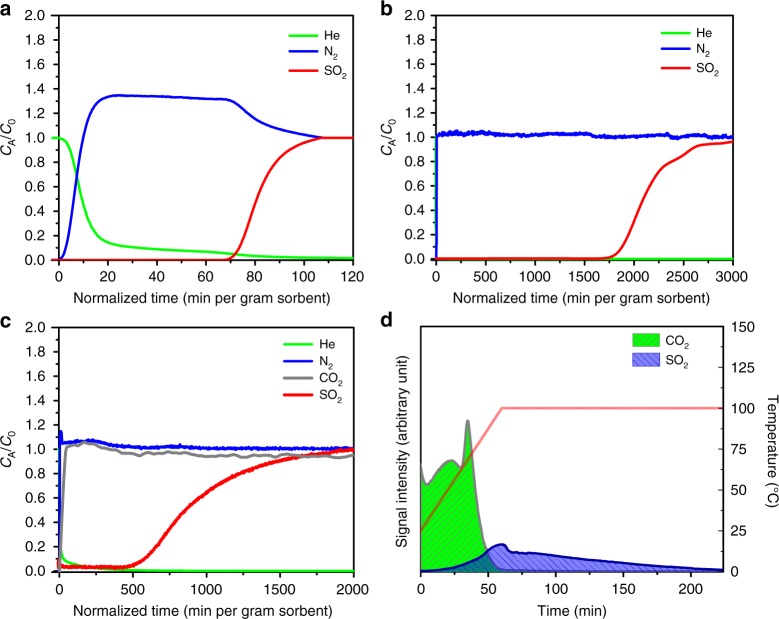
CO_2_/SO_2_ capture performance of KAUST**-**8. Adsorption column breakthrough experiments for KAUST**-**8 with **a** SO_2_/N_2_: 7/93 (10 cm^3^ min^−1^, flow rate), **b** SO_2_/N_2_: 0.05/99.95 mixture (40 cm^3^ min^−1^, flow rate), and **c** SO_2_/CO_2_/N_2_: 0.05/10/89.95 (40 cm^3^ min^−1^, flow rate). **d** TPD experiment suggests a considerable amount of SO_2_ along with CO_2_ as adsorbed phase after a breakthrough experiment with 500 p.p.m. SO_2_ in the presence of 10% CO_2_ and balance N_2_

Investigation of single component SO_2_ adsorption showed that KAUST-8 also exhibits steep adsorption isotherm at 25 °C (Supplementary Figure 10). The corresponding adsorption column breakthrough experiment with SO_2_/N_2_: 7/93 mixture showed a higher uptake of 2.2 mmol g^−1^ (Fig. 4a). KAUST-8 can be completely regenerated by heating at 105 °C in a vacuum or inert gas environment (Supplementary Figure 11), confirming SO_2_ stability and recyclability. During the adsorption column breakthrough experiments carried out with low SO_2_ (SO_2_/N_2_: 0.05/99.95) mixture, KAUST-8 still maintained a high uptake of SO_2_ (1.6 mmol g^−1^) (Fig. 4b, Supplementary Table 3). Subsequent TPD analysis of the adsorbed phase confirmed the adsorption of SO_2_ (Supplementary Figure 12) at p.p.m. level. Supplementary Table 3 compares SO_2_ uptake of different benchmark MOFs at low concentration. Materials with relatively more open structure such as MFM-300(In, Al) have very high SO_2_ uptake at higher SO_2_ concentration; however, they have lower SO_2_ uptake at lower concentrations close to 500 p.p.m. (of interest for application such as SO_2_ capture from flue gas and SO_2_ sensing) compared with KAUST-7, KAUST-8, and some SIF_6_^2−^ based MOFs. Materials such as MFM-300(In, Al) with very high SO_2_ uptake at higher SO_2_ concentration could have many important applications. MFM-300(In, Al) (very high uptake at higher concentration), and KAUST-7 and KAUST-8 (high uptake at very low concentration) are complementary to each other and their uses depend upon nature of the application. Adsorption column breakthrough experiments with synthetic flue gas using a SO_2_/CO_2_/N_2_: 0.05/10/89.95 mixture showed that SO_2_ continues to be adsorbed for long durations past the CO_2_ breakthrough time (Fig. 4c). This indicates that the adsorbed CO_2_ is replaced by SO_2_ from the gas mixture, which is consistent with a much higher estimated interaction energy of SO_2_ over CO_2_. Subsequent TPD analysis suggests an adsorbed phase composition of 1.5 mmol g^−1^ for CO_2_ and 0.5 mmol g^−1^ for SO_2_, which is remarkable considering the large difference in concentrations of CO_2_ and SO_2_ in the synthetic flue gas (Fig. 4d). A selectivity of SO_2_/CO_2_ ≈ 66 obtained from the combination of breakthrough and TPD experiment shows that KAUST-8 is a highly efficient material for SO_2_ removal at a p.p.m. level and is promising for selectively removing SO_2_ from flue gas. The performance of KAUST-8 using humid (≈40% RH) 250 p.p.m. SO_2_/ balance N_2_ gas mixture was also investigated (Supplementary Figure 13). For KAUST-8, also SO_2_ has almost the same breakthrough time as that of water; however, owing to its higher water adsorbing capacity than KAUST-7, it can adsorb more SO_2_ compared with KAUST-7 under the same conditions. Uptake of SO_2_ by KAUST-8 in humid condition is notable considering in the above experiment gas stream contained almost 50 times higher concentration of water than SO_2_. In addition to previously reported excellent water stability^46^, SO_2_ stability of the KAUST-8 was proven by PXRD comparison of the materials before and after dry and humid SO_2_ exposure (Supplementary Figure 14), CO_2_ breakthrough study before and after 7% SO_2_ breakthrough experiments (Supplementary Figure 15) and CO_2_ adsorption isotherm after the humid SO_2_ breakthrough experiment (Supplementary Figure 16).

### Selective SO_2_ detection from air

From the adsorptive separation study above, KAUST-8 and KAUST-7 were shown to exhibit tunable CO_2_/H_2_S selectivity, molecules that are present in environments contaminated with SO_2_. To benefit from the outstanding properties of this platform, we found it compelling to explore the feasibility of depositing KAUST-8 and KAUST-7 on a QCM electrode and unveiling their SO_2_ sensing properties in the presence and absence of humidity to mimic atmospheric conditions. Weight-detectable sensor such as QCM has been used in this study to detect SO_2_ accurately when the disturbing presence of moisture is involved. These imply that QCM device can be an alternative to IDE^48^ when testing for a gas in the presence of humidity. Part of the reason for this difference is that IDE sensors are based on the change in dielectric constant (*ε*_H2O_ = 80 and *ε*_SO2_ = 16) and in order to detect SO_2_ accurately in the presence of moisture the deposited materials on the IDE-type electrode has to overcome the high dielectric constant for H_2_O.

**Fig. 5:**
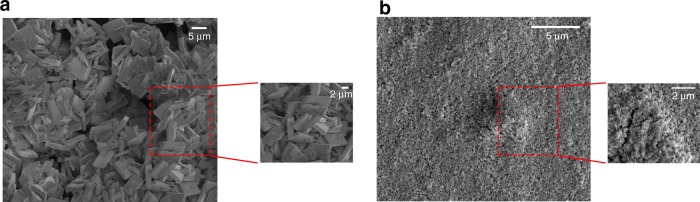
Scanning electron microscopy of thin films. SEM micrographs at a high and low magnification of **a** KAUST-7 and **b** KAUST-8 thin films coated on the gold electrode of QCM

The surface morphology of KAUST-8 and KAUST-7 coated on QCM (see inset) was studied using scanning electron microscopy. The thin films of both MOFs were found to be compact and uniform. The densely packed MOFs crystals were uniformly deposited on the QCM substrate with low intergranular voids and random orientation. As illustrated in Fig. 5, the coating of KAUST-7 led to cubic crystallites of ~150 nm, whereas for the KAUST-8 films the size of the crystallites is significantly larger at ∼30 μm. PXRD experiments were carried out to confirm the purity and crystallinity of the deposited MOFs (Supplementary Figure 17).

**Fig. 6:**
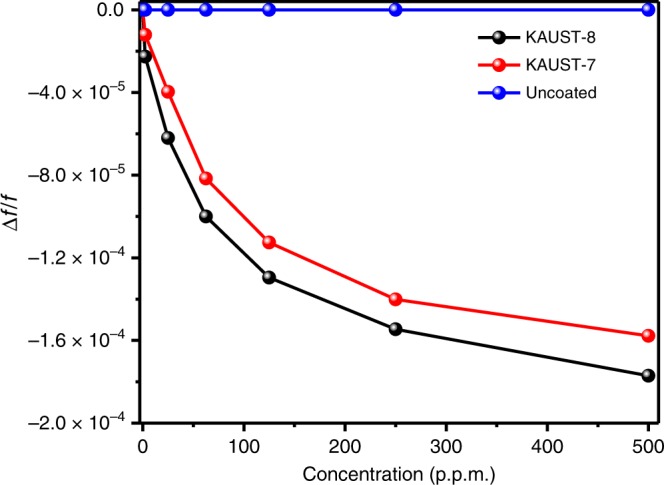
Frequency shift comparisons. Frequency shift as a function of the SO_2_ concentration for the uncoated and KAUST-7- or KAUST-8-coated QCM sensors

The sensitivity (∆*f*/*f*)) of KAUST-8- and KAUST-7-coated QCM devices were measured for different concentrations of SO_2_, ranging from 0 to 500 p.p.m. in balance with nitrogen^18^. Uncoated QCM showed a negligible response to SO_2_. With the increase in the concentration of SO_2_, both MOF-coated sensors responded with a nonlinear decrease in sensitivity (Fig. 6) and (Supplementary Figure 18). The lowest detection limit, defined theoretically using a largely accepted methodology by the sensing community^42^, was estimated to be about 100 p.p.b. (with noise drift in the resonance frequency of ± 1.5 Hz)^49^. By optimizing the device parameters, drift in the sensors resonance frequency, this detection limit can be reduced to below 0.2 Hz^50,51^ and thereby improving the detection limit to < 15 p.p.b. This discrepancy/gap between the theoretical and experimental detection limits (15 p.p.b. and 5 p.p.m.) was never discussed previously in the open literature.

After each exposure cycle, the device was in situ heated at 105 °C in ambient nitrogen for 4 h, allowing the reactivation of the evaluated MOF thin film for another sensing cycle.

**Fig. 7:**
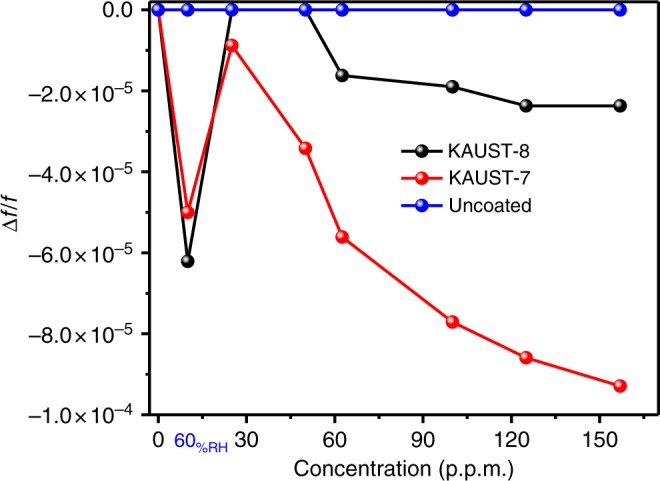
Sensor responses. Plots of sensors responses as a function of SO_2_ concentration in synthetic air

Humidity is present in most environments, and so it is important to understand a sensors response in its presence. Therefore, mixed gas experiments were performed, exposing KAUST-7 and KAUST-8 to SO_2_ in humid conditions mimicking real-world conditions for SO_2_ detection. Figure 7 shows the sensor sensitivity as a function of SO_2_ concentration in humid conditions (60% RH) at RT for uncoated and coated KAUST-7 and KAUST-8 QCMs. Uncoated QCM has a near-zero response to humidity and SO_2_. This corroborates that the sensing response to SO_2_ under humid conditions is due to its affinity to KAUST-7 and KAUST-8 films.

The responses of the two kinds of sensors were different. As seen in Fig. 7 and (Supplementary Figure 19), the resonance frequency of the QCMs initially decreased when the ambience was changed from dry to humid SO_2_ conditions. The most prominent difference is the inversion in the sensor output due to the introduction of SO_2_ at 60% RH but not in the same manner as compared with the dry SO_2_ case. Interestingly, when exposed to 25 p.p.m. of SO_2_ in the above-mentioned humid conditions, the sensor resonance frequency for SO_2_ was reduced. Under humid conditions, the sensitivity of the two MOFs slightly reduced when compared with dry conditions. However, KAUST-7 films demonstrated a four-time higher sensitivity toward SO_2_ in the presence of humidity compared with KAUST-8.

To further analyze the results obtained, it is necessary to consider the specific features of the adsorption of SO_2_ and water on the surface of KAUST-7 and KAUST-8. As seen in Fig. 7, the presence of humidity (60% RH) did not significantly affect the KAUST-7-based sensors response to the SO_2_ analyte. This may be due to the affinity of SO_2_ molecules to replace some of the adsorbed water molecules or/and coexist in the highly confined pore system. In the case of KAUST-8-based sensor, which is isomorphic to the KAUST-7, lower sensitivity to SO_2_ in the presence of humidity was observed. Although SO_2_ has the affinity to replace water molecules, the reduced sensitivity is attributed to the absence of accessible ultra-microporous morphology. The number of SO_2_-adsorbing active sites is reduced by the pre-adsorbed water, thereby limiting the available space in the pore system for adsorption. This observation is supported by the fact that the water molecules strongly interact with Al^3+^^46^ with higher host/guest interaction energy as compared with SO_2_. The TPD experiment results (Supplementary Figure 20) show that in the case of KAUST-7, the SO_2_ can replace the already adsorbed water molecules relatively easy as compared with the KAUST-8, which has stronger water affinity. These results are in agreement with the conclusion reported in the literature for the simultaneous capture of CO_2_ and H_2_O using KAUST-8^46^.

**Fig. 8:**
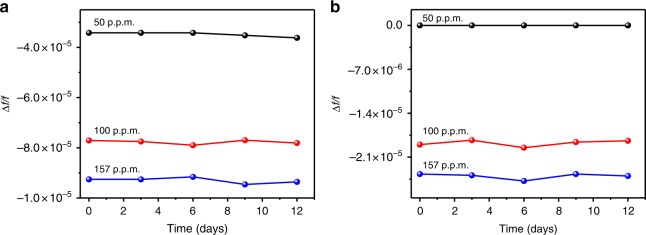
Stability and reproducibility. Long-term stability property of the **a** KAUST-7 and **b** KAUST-8 sensors exposed to 50, 100, and 157 p.p.m. SO_2_ gas

The most important parameters for a sensing device are its stability and reproducibility. These parameters were investigated by cyclic exposure of the sensor to different SO_2_ concentrations after every 48 h at RT over a period of 12 days (Fig. 8). The three results demonstrated the stability of the sensors exposed to 50, 100, and 157 p.p.m. SO_2_ gas with no significant change in the resonant frequency over time.

## Discussion

In this report, we successfully demonstrated the superior performance of two fluorinated MOFs, namely KAUST-7 and KAUST-8, for the capture of SO_2_ from flue gas. Combined mixed gas breakthrough experiments and molecular simulation confirmed that simultaneous capture of SO_2_ and CO_2_ occurs using KAUST-7, whereas KAUST-8 displays a higher affinity for SO_2_ with SO_2_/CO_2_ selectivity ≈66. Based on this performance, QCM-based sensors were successfully fabricated for sensing SO_2_ from air using this fluorinated MOF platform (Supplementary Figure 21). Both MOF materials confirmed their potential, revealing good SO_2_ detection capabilities above 25 p.p.m., the range of SO_2_ concentrations in the air-inducing nose and eye irritation. This remarkable performance of sensing makes these materials highly desirable for the fabrication of new advanced devices to improve health and environmental conditions.

## Methods

### Materials

All reagents were used as received from commercial suppliers without further purification.

KAUST-7^44,45^ and KAUST-8^46^ were synthesized according to literature procedures.

### Powder X-ray diffraction

The PXRD data were collected over the 2*θ* range 4–40° or 6–80° on a high-resolution PANalytical X’Pert MPD-PRO diffractometer with Cu *K*α_1_ radiation (*λ* = 1.5406 Å, 45 kV/40 mA).

### Single crystal X-ray diffraction

The SCXRD data were collected using a Bruker X8 PROSPECTOR APEX2 CCD diffractometer (Cu *K*α, *λ* = 1.54178 Å). Indexings were performed using APEX2 (Difference Vectors method)^52^. Data integration and reduction were performed using SaintPlus^53^. Absorption corrections were performed by multi-scan method implemented in SADABS^54^. Space groups were determined using XPREP implemented in APEX2^52^. Both structures were solved using Direct Methods (SHELXS-97) and refined using SHELXL-2018/3 (full-matrix least-squares on *F*^2^) contained WinGX v1.70.01^55–57^.

### In situ gas loading experiment

We used commercially available Swagelok miniature quick connect with a small modification that allowed us to fix 0.3 mm glass capillary loaded with crystal above the quick connect. The selected single crystal was glued to glass fiber and placed in 0.3 mm glass capillary before attaching it to quick connect. In situ pretreatment of the crystal loaded in environmental gas cell was carried out first under dry nitrogen flow. Temperature of the crystal increased by immersing the capillary in to oil bath. Temperature of the oil bath was fixed at 140 °C for both the samples to obtain 105–120 °C effective temperature at the crystal. After 24 h, nitrogen flow was changed to dynamic vacuum keeping the temperature constant. After 12 h, crystal was allowed to cool down under dynamic vacuum and then 7% SO_2_ (93% N_2_) was introduced into environmental cell at 4–5 bar pressure. In case of CO_2_, 100% CO_2_ was introduced at 2–3 bar. The gas-loaded environmental cell was detached under pressure and allowed to equilibrate for around 8 h. After 8 h, the environmental cell was mounted on a modified goniometer for single crystal X-ray data collection.

### Column breakthrough test set-up, procedure, and measurements

The experimental setup used for dynamic breakthrough measurements is shown in Supplementary Figure 22. The gas manifold consisted of three lines fitted with mass flow controllers. Line A is used to feed an inert gas, most commonly helium, to activate the sample before each experiment. The other two lines B and C feed a pure or pre-mixed gases. Whenever required, gases flowing through lines B and C may be mixed before entering a column packed with the sample using a four-way valve. In a typical experiment, 300–500 mg of adsorbent (in the column) was treated in situ at a required temperature under He flow (50 cm^3^ g^−1^) for 8 h.

Before starting each experiment, helium reference gas is flushed through the column and then the gas flow is switched to the desired gas mixture at the same flow rate between10–50 cm^3^ g^−1^. The gas mixture downstream the column was monitored using a Hiden mass spectrometer.

### Fabrication of KAUST-7- and KAUST-8-coated QCM

The transducer was a 10 MHz AT-cut piezoelectric QCM device with a thickness shear mode and placed between two gold electrodes for electrical connection. The QCM is rinsed with ethanol and dried in air. MOFs paste was then applied to the electrode of QCMs by spin-coating method (2 μm thick); no prior modification of the sensors surface was required.

The QCM sensor was then fixed in a sealed chamber. Before measurements, the fresh coated MOFs film was activated in situ for 4 h to have a guest free framework. The resulting coatings are ultrathin and reproducible so that the stress upon absorption of SO_2_ inducing a change in the mass change of the thin film is effective.

### Sensing apparatus

Supplementary Figure 23 shows the sensing setup used in this study for real-time measurement. All the sensor measurements were carried out at RT, under a dry air total stream of 200 cm^3^ m^−1^. MFCs (mass flow controllers) from Alicat Scientific, Inc. were used to control the flow rate for gases coming from certified bottles. Stainless steel delivery lines or perfluoroalkoxy alkane, Perfluoroalkoxy (PFA) tubing (in regions requiring flexibility and resistivity to volatile organic compounds (VOCs)) were used on the setup with Vernier metering valves (from Swagelok) as a flow regulator. To detect the change in humidity level inside the chamber, a commercial humidity sensor (Honeywell HIH-4000–003) was used as a reference, which has an error < 0.5% RH. The QCM sensor was exposed to the analyte stream until a stable response was obtained; a two-port network (Keysight E5071C ENA) circuit was used to monitor the change in resonance frequency. A LabVIEW interface was used for synchronization and data acquisition by controlling the LCR meter (L = Inductance, C = Capacitance, R = Resistance) and the multimeter. Hence, the possibility of data loss was minimized.

### Computational methods

All simulations were performed using the codes available in the Quantum Espresso package^58^, which implements the DFT under periodic boundary conditions with planewave functions as basis sets^59^. The geometry optimizations were performed with the generalized gradient approximation (Perdew-Burke-Ernzerhof (PBE))^60^ including the dispersion corrections described by the Grimme’s scheme^61^, maintaining fixed the atomic coordinates of the F-Ni-F chains and the unit cell dimensions. The relative ion positions were relaxed until all of the force components were lower than 0.001 Ry Bohr^−1^. The equilibrium atomic positions of all systems were found by minimizing the total energy gradient. The ion cores were described by Vanderbilt ultrasoft pseudopotential^62^ and the Kohn–Sham one-electron states were expanded in a planewave basis set with a kinetic cutoff energy of 50 Ry (500 Ry for the density) All the calculations were performed at the Γ-point. The Hubbard approach was also applied in order to better describe the localized *d* states of nickel. The Hubbard parameters (*U*) were taken from the calculations of Wang et al.^63^, with a value of 6.4 eV for nickel. The Marzari–Vanderbilt smearing technique was used^64^ with a broadening of 0.005 Ry in order to smooth the Fermi distribution.

The geometry optimizations of SO_2_ and CO_2_ in both MOFs were performed by considering one guest molecule per formula unit. The interaction energy was then calculated using the following equation: Δ*E* = *E*_MOF/guest molecule_ − *E*_guest molecule_ − *E*_empty MOF_, where *E*_MOF/guest molecule_, *E*_guest molecule_, and *E*_empty MOF_ are the total energy of the loaded system (guest molecule + MOF), of the guest molecule and the single-point total energy of the MOF, respectively.

## Supplementary information

Supplementary Information

## Data Availability

The X-ray crystallographic data for KAUST-7 (SO_2_) and KAUST-8 (SO_2_) have been deposited at the Cambridge Crystallographic Data Centre (CCDC), under deposition numbers 1871683 and 1871684, respectively. These data can be obtained free of charge from the CCDC via www.ccdc.cam.ac.uk. All other relevant data supporting the findings of this study are available from the corresponding authors on request.

## References

[1] Adil K (2017). Valuing metal–organic frameworks for postcombustion carbon capture: a benchmark study for evaluating physical adsorbents. Adv. Mater..

[2] Rezaei F, Rownaghi AA, Monjezi S, Lively RP, Jones CW (2015). SO_x_/NO_x_ removal from flue gas streams by solid adsorbents: a review of current challenges and future directions. Energy Fuels.

[3] Ryu HJ, Grace JR, Lim CJ (2006). Simultaneous CO_2_/SO_2_ capture characteristics of three limestones in a fluidized-bed reactor. Energy Fuels.

[4] Clarke AG, Radojevic M (1987). Oxidation of SO_2_ in rainwater and its role in acid rain chemistry. Atmos. Environ. (1967).

[5] Galloway JN, Dianwu Z, Jiling X, Likens GE (1987). Acid rain: China, United States, and a remote area. Science.

[6] Berger F, Fromm M, Chambaudet A, Planade R (1997). Tin dioxide-based gas sensors for SO_2_ detection: a chemical interpretation of the increase in sensitivity obtained after a primary detection. Sens. Actuators B Chem..

[7] Torvela H, Huusko J, Lantto V (1991). Reduction of the interference caused by NO and SO_2_ in the CO response of Pd-catalysed SnO_2_ combustion gas sensors. Sens. Actuators B Chem..

[8] Shimizu Y, Matsunaga N, Hyodo T, Egashira M (2001). Improvement of SO_2_ sensing properties of WO_3_ by noble metal loading. Sens. Actuators B Chem..

[9] Stankova M (2004). Detection of SO_2_ and H_2_S in CO_2_ stream by means of WO3-based micro-hotplate sensors. Sens. Actuators B Chem..

[10] Penza M, Cassano G, Tortorella F (2001). Gas recognition by activated WO_3_ thin-film sensors array. Sens. Actuators B Chem..

[11] Gardon M, Guilemany JM (2013). A review on fabrication, sensing mechanisms and performance of metal oxide gas sensors. J. Mat. Sci. Mater. Electron..

[12] Barsan N, Koziej D, Weimar U (2007). Metal oxide-based gas sensor research: How to?. Sens. Actuators B Chem..

[13] Williams DE (1999). Semiconducting oxides as gas-sensitive resistors. Sens. Actuators B Chem..

[14] Korotcenkov G (2007). Metal oxides for solid-state gas sensors: what determines our choice?. Mater. Sci. Eng. B.

[15] Hübert T, Boon-Brett L, Black G, Banach U (2011). Hydrogen sensors – a review. Sens. Actuators B Chem..

[16] Yan Y, Wladyka C, Fujii J, Sockanathan S (2015). Prdx4 is a compartment-specific H_2_O_2_ sensor that regulates neurogenesis by controlling surface expression of GDE2. Nat. Commun..

[17] Awang Z (2014). Gas sensors: a review. Sens. Transducers.

[18] Chappanda KN (2018). The quest for highly sensitive QCM humidity sensors: the coating of CNT/MOF composite sensing films as case study. Sens. Actuators B Chem..

[19] Li JR, Kuppler RJ, Zhou HC (2009). Selective gas adsorption and separation in metal–organic frameworks. Chem. Soc. Rev..

[20] Bhatt PM (2017). Isoreticular rare earth fcu-MOFs for the selective removal of H_2_S from CO_2_ containing gases. Chem. Eng. J..

[21] Cui X (2017). Ultrahigh and selective SO_2_ uptake in inorganic anion‐pillared hybrid porous materials. Adv. Mater..

[22] Kreno LE (2011). Metal–organic framework materials as chemical sensors. Chem. Rev..

[23] Mohideen MIH (2017). A fine-tuned MOF for gas and vapor separation: a multipurpose adsorbent for acid gas removal, hehydration, and BTX sieving. Chem.

[24] AbdulHalim RG (2017). A fine-tuned metal–organic framework for autonomous indoor moisture control. J. Am. Chem. Soc..

[25] Tansell AJ, Jones CL, Easun TL (2017). MOF the beaten track: unusual structures and uncommon applications of metal–organic frameworks. Chem. Cent. J..

[26] Adil K (2017). Gas/vapour separation using ultra-microporous metal–organic frameworks: insights into the structure/separation relationship. Chem. Soc. Rev..

[27] Yang S (2012). Selectivity and direct visualization of carbon dioxide and sulfur dioxide in a decorated porous host. Nat. Chem..

[28] Mathew S (2016). Selective adsorption of sulfur dioxide in a robust metal–organic framework material. Adv. Mater..

[29] Rodríguez-Albelo LM (2017). Selective sulfur dioxide adsorption on crystal defect sites on an isoreticular metal organic framework series. Nat. Commun..

[30] Rad AS, Chourani A (2017). Nickel based paddle-wheel metal–organic frameworks towards adsorption of O_3_ and SO_2_ molecules: quantum-chemical calculations. J. Inorg. Organomet. Polym. Mater..

[31] Lee GY (2017). Amine-functionalized covalent organic framework for efficient SO_2_ capture with high reversibility. Sci. Rep..

[32] Glomb S, Woschko D, Makhloufi G, Janiak C (2017). Metal–organic frameworks with internal urea-functionalized dicarboxylate linkers for SO_2_ and NH_3_ adsorption. ACS Appl. Mater. Interfaces.

[33] Fernandez CA (2010). Gas-induced expansion and contraction of a fluorinated metal−organic framework. Cryst. Growth Des..

[34] Tan K (2013). Mechanism of preferential adsorption of SO_2_ into two microporous paddle wheel frameworks M(bdc)(ted)_0.5_. Chem. Mater..

[35] Thallapally PK, Motkuri RK, Fernandez CA, McGrail BP, Behrooz GS (2010). Prussian blue analogues for CO_2_ and SO_2_ capture and separation applications. Inorg. Chem..

[36] Yang S (2013). Irreversible network transformation in a dynamic porous host catalyzed by sulfur dioxide. J. Am. Chem. Soc..

[37] Zhang D, Wu J, Li P, Cao Y (2017). Room-temperature SO_2_ gas-sensing properties based on a metal-doped MoS_2_ nanoflower: an experimental and density functional theory investigation. J. Mat. Chem. A.

[38] Zhang D, Liu J, Jiang C, Li P (2017). High-performance sulfur dioxide sensing properties of layer-by-layer self-assembled titania-modified graphene hybrid nanocomposite. Sens. Actuators B: Chem..

[39] Sasaki I, Tsuchiya H, Nishioka M, Sadakata M, Okubo T (2002). Gas sensing with zeolite-coated quartz crystal microbalances—principal component analysis approach. Sens. Actuators B: Chem..

[40] Osada M, Sasaki I, Nishioka M, Sadakata M, Okubo T (1998). Synthesis of a faujasite thin layer and its application for SO_2_ sensing at elevated temperatures. Microporous Mesoporous Mater..

[41] Lee SC (2011). A novel tin oxide-based recoverable thick film SO_2_ gas sensor promoted with magnesium and vanadium oxides. Sens. Actuators B Chem..

[42] Chernikova V, Yassine O, Shekhah O, Eddaoudi M, Salama KN (2018). Highly sensitive and selective SO_2_ MOF sensor: the integration of MFM-300 MOF as a sensitive layer on a capacitive interdigitated electrode. J. Mater. Chem. A.

[43] Sapsanis C (2015). Insights on capacitive interdigitated electrodes coated with MOF thin films: Humidity and VOCs sensing as a case study. Sensors.

[44] Bhatt PM (2016). A fine-tuned fluorinated MOF addresses the needs for trace CO_2_ removal and air capture using physisorption. J. Am. Chem. Soc..

[45] Cadiau A, Adil K, Bhatt P, Belmabkhout Y, Eddaoudi M (2016). A metal-organic framework–based splitter for separating propylene from propane. Science.

[46] Cadiau A (2017). Hydrolytically stable fluorinated metal-organic frameworks for energy-efficient dehydration. Science.

[47] Belmabkhout, Y. et al. Natural gas upgrading using a fluorinated MOF with tuned H2S and CO2 adsorption selectivity. *Nature Energ*y **3**, 1059–1066 (2018).

[48] Zeinali, S., Homayoonnia, S. & Homayoonnia, G. Comparative investigation of interdigitated and Parallel-plate capacitive gas sensors based on Cu-BTC nanoparticles for selective detection of polar and apolar VOCs indoors. *Sens. Actuators B Chem*. **278**, 153–164 (2018).

[49] Yamagiwa H (2014). Detection of volatile organic compounds by weight-detectable sensors coated with metal-organic frameworks. Sci. Rep..

[50] Rodriguez-Pardo L, Rodríguez JF, Gabrielli C, Perrot H, Brendel R (2005). Sensitivity, noise, and resolution in QCM sensors in liquid media. IEEE Sens. J..

[51] Cao-Paz AM, Rodríguez-Pardo L, Fariña J, Marcos-Acevedo J (2012). Resolution in QCM sensors for the viscosity and density of liquids: application to lead acid batteries. Sensors.

[52] APEX2 Ver. 2014.11-0, Bruker AXS, Inc., Madison, Wisconsin, USA, 2014.

[53] SAINT Ver.8.34A. Bruker AXS, Inc., Madison, Wisconsin, USA, 2014.

[54] SADABS Ver. 2014/15. Bruker AXS, Inc., Madison, Wisconsin, USA, 2014

[55] SHELXS-97, Program for Crystal Structure Solution (Univ. Göttingen, Germany, 1997).

[56] Sheldrick, G.M. SHELXL-2018/3. Crystal structure refinement with SHELXL. *Acta Cryst*. **C71**, 3–8 (2015). 10.1107/S2053229614024218 PMC4294323 25567568

[57] WinGX. Farrugia LJ (2012). WinGX and ORTEP for Windows: an update. J. Appl. Cryst..

[58] Giannozzi P (2009). QUANTUM ESPRESSO: a modular and open-source software project for quantum simulations of materials. J. Phys. Condens. Matter.

[59] Hohenberg P, Kohn W (1964). Inhomogeneous electron gas. Phys. Rev..

[60] Kohn W, Sham LJ (1965). Self-consistent equations including exchange and correlation effects. Phys. Rev..

[61] Perdew JP, Burke K, Ernzerhof M (1996). Generalized gradient approximation made simple. Phys. Rev. Lett..

[62] Vanderbilt D (1990). Soft self-consistent pseudopotentials in a generalized eigenvalue formalism. Phys. Rev. B.

[63] Wang L, Maxisch T, Ceder G (2006). Oxidation energies of transition metal oxides within the GGA+ U framework. Phys. Rev. B.

[64] Marzari N, Vanderbilt D, De Vita A, Payne M (1999). Thermal contraction and disordering of the Al (110) surface. Phys. Rev. Lett..

