# Supplementary material for: Fluorinated MOF platform for selective removal and sensing of SO2 from flue gas and air
Source: Nat Commun. 2019 Mar 22;10:1328. doi: 10.1038/s41467-019-09157-2 (PMC6430820; doi:10.1038/s41467-019-09157-2)
Supplement: Supplementary file 1 — Supplementary Information [file 41467_2019_9157_MOESM1_ESM.pdf]

Supplementary Information

Fluorinated MOF Platform for selective removal and sensing of SO_2 from flue gas and air

M. R. Tchalala et. al.

Supplementary Figures

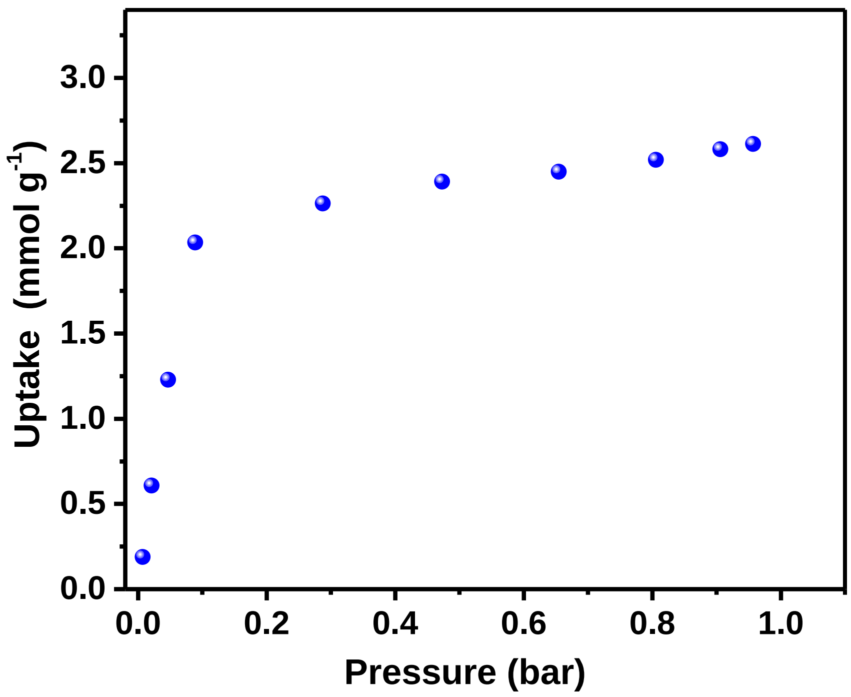

Supplementary Figure 1. SO_2 isotherm for KAUST-7 at 25 °C after 105 °C activation.

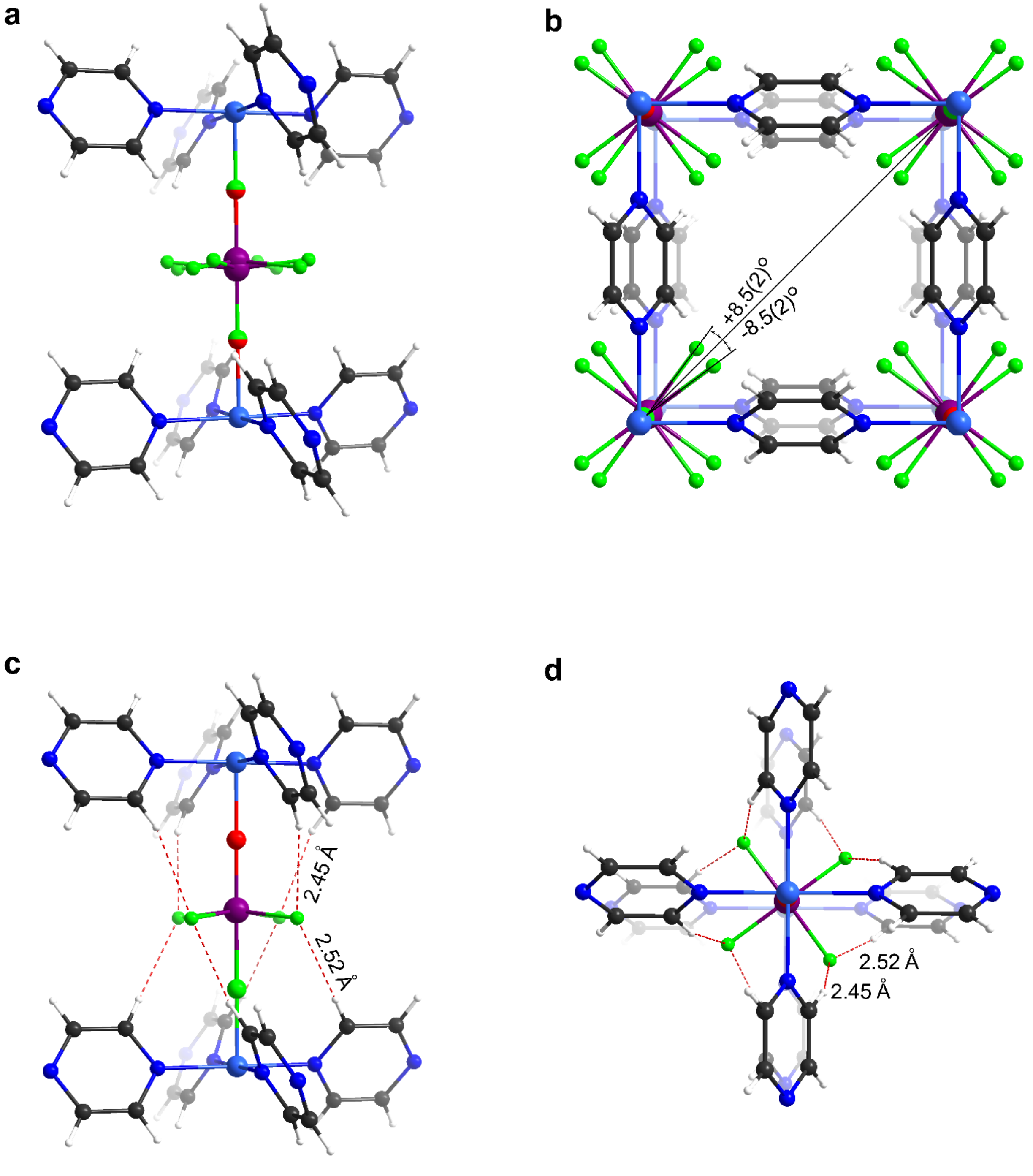

Supplementary Figure 2. KAUST-7 (SO_2) crystal structure: a) axial disorder of $(NbOF_5)^{2-}$ anion; b) $(NbOF_5)^{2-}$ twist from the diagonal direction; Pyrazine-pillar C–H···F hydrogen bonding side c) and top d) view. Only one orientation of $(NbOF_5)^{2-}$ is shown.

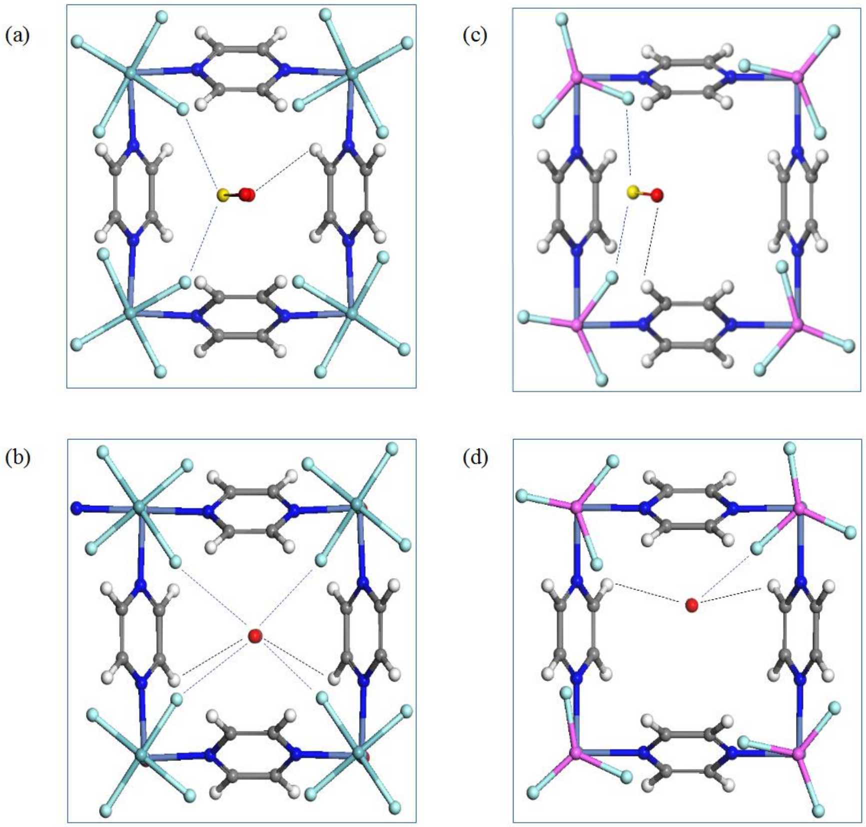

Supplementary Figure 3. Local views for the DFT-geometry optimized guest loaded-crystal structures: (a) SO_2 and (b) CO_2- loaded in KAUST-7, (c) SO_2 and (d) CO_2- loaded in KAUST-8. Color code: Aluminum (pink), niobium (green), nickel (steel blue), fluorine (light green), nitrogen (blue), carbon (gray), hydrogen (white), oxygen (red), and sulfur (yellow).

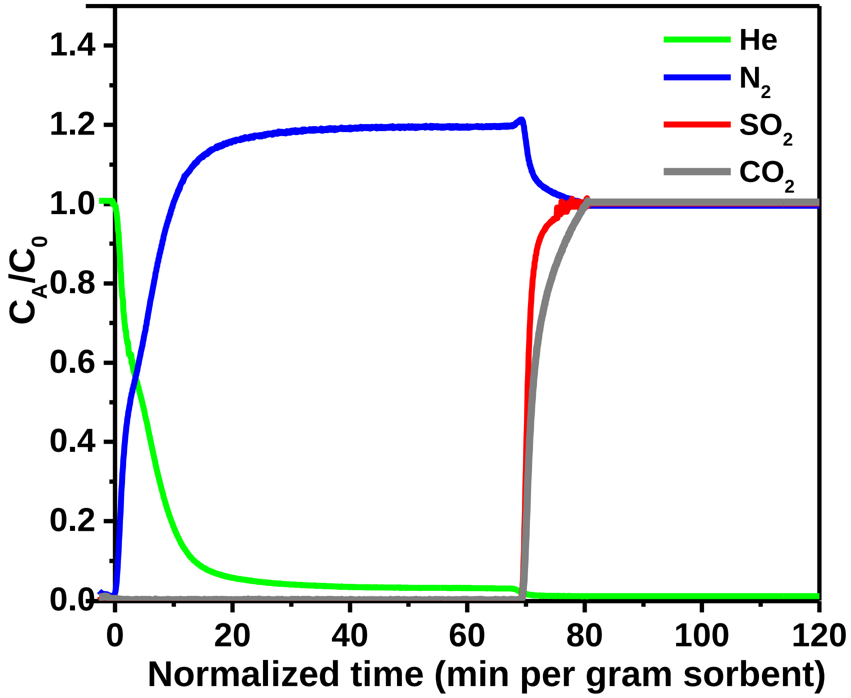

Supplementary Figure 4. KAUST-7 breakthrough experiments with 4% CO_2, 4% SO_2 (balance N_2) gas mixture feed (10.cc/min) at 298 K and 1 bar.

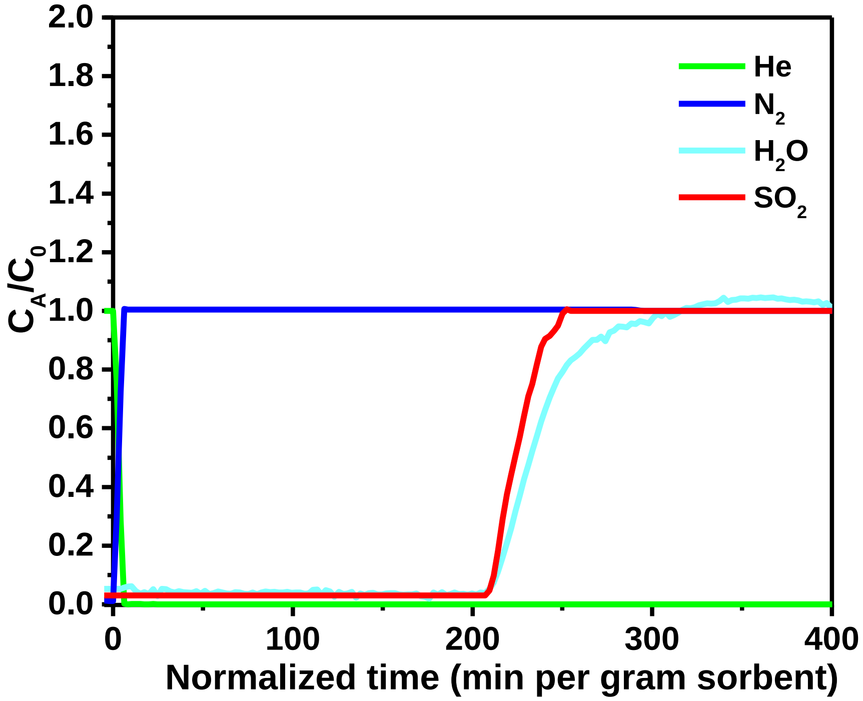

Supplementary Figure 5. Breakthrough experiment for KAUST-7 using humid (≈40% RH) 250 ppm SO_2/ balance N_2 gas mixture feed at the flow rate of 50cc/min (1bar, 298 K).

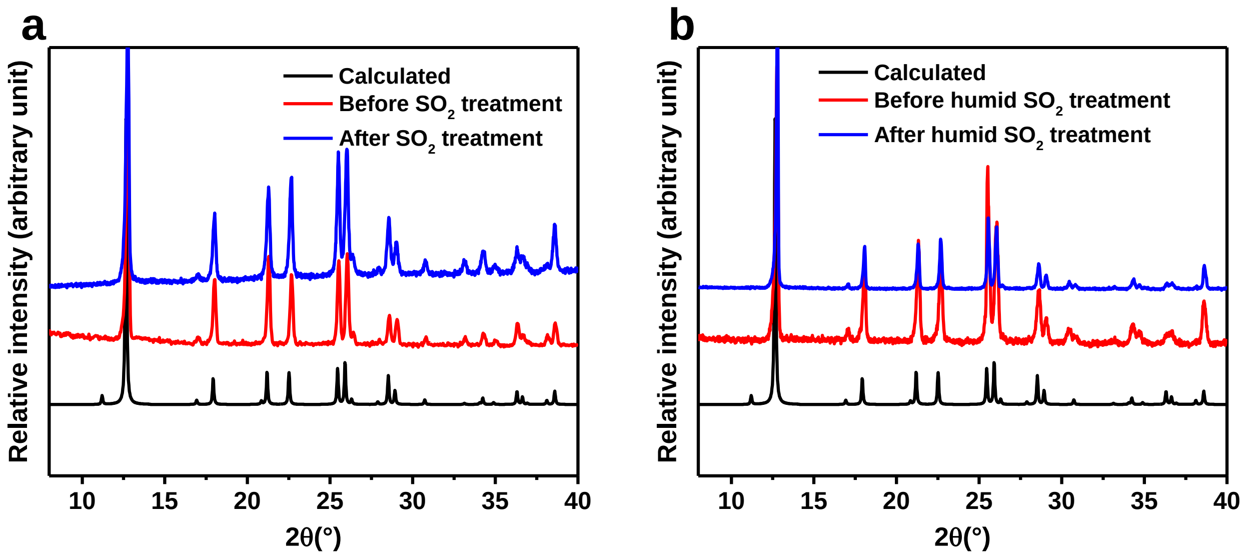

Supplementary Figure 6. (a) Comparison of PXRD patterns of KAUST-7 before (red) and after SO_2 exposure (blue) with calculated PXRD (black) from the single crystal structure, **(b)** Comparison of PXRD patterns of KAUST-7 before (red) and after humid SO_2 exposure (blue) with calculated PXRD (black) from the single crystal structure,

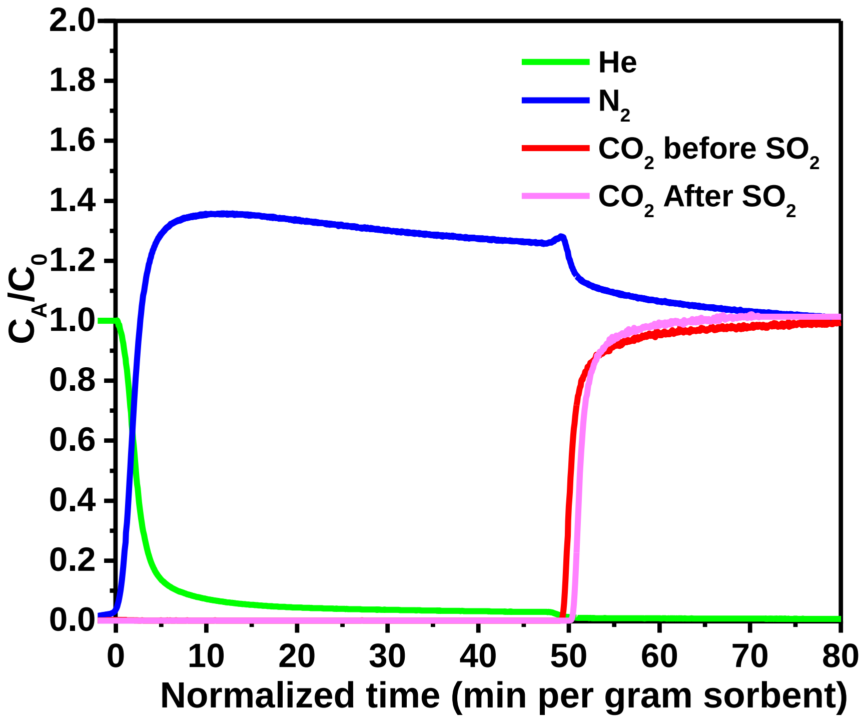

Supplementary Figure 7. Breakthrough experiments for KAUST-7 using 10% CO_2/balance nitrogen feed (10,cc/min) before and after 7% SO_2 (balance N_2) breakthrough experiments.

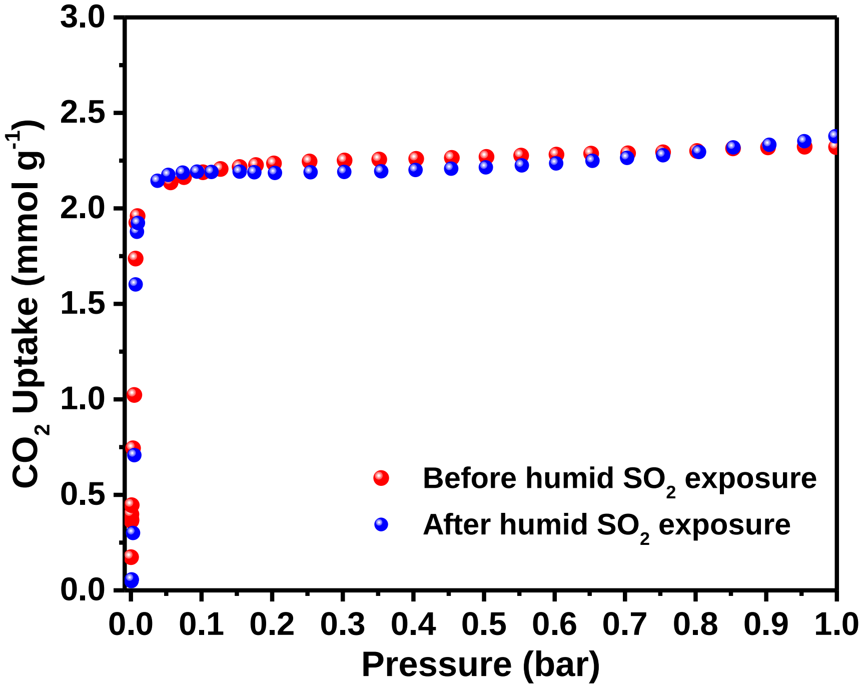

Supplementary Figure 8. CO_2 isotherms at 298 K for KAUST-7 before and after a breakthrough experiment with humid (≈40% RH) 250 ppm SO_2/ balance N_2 gas mixture.

.

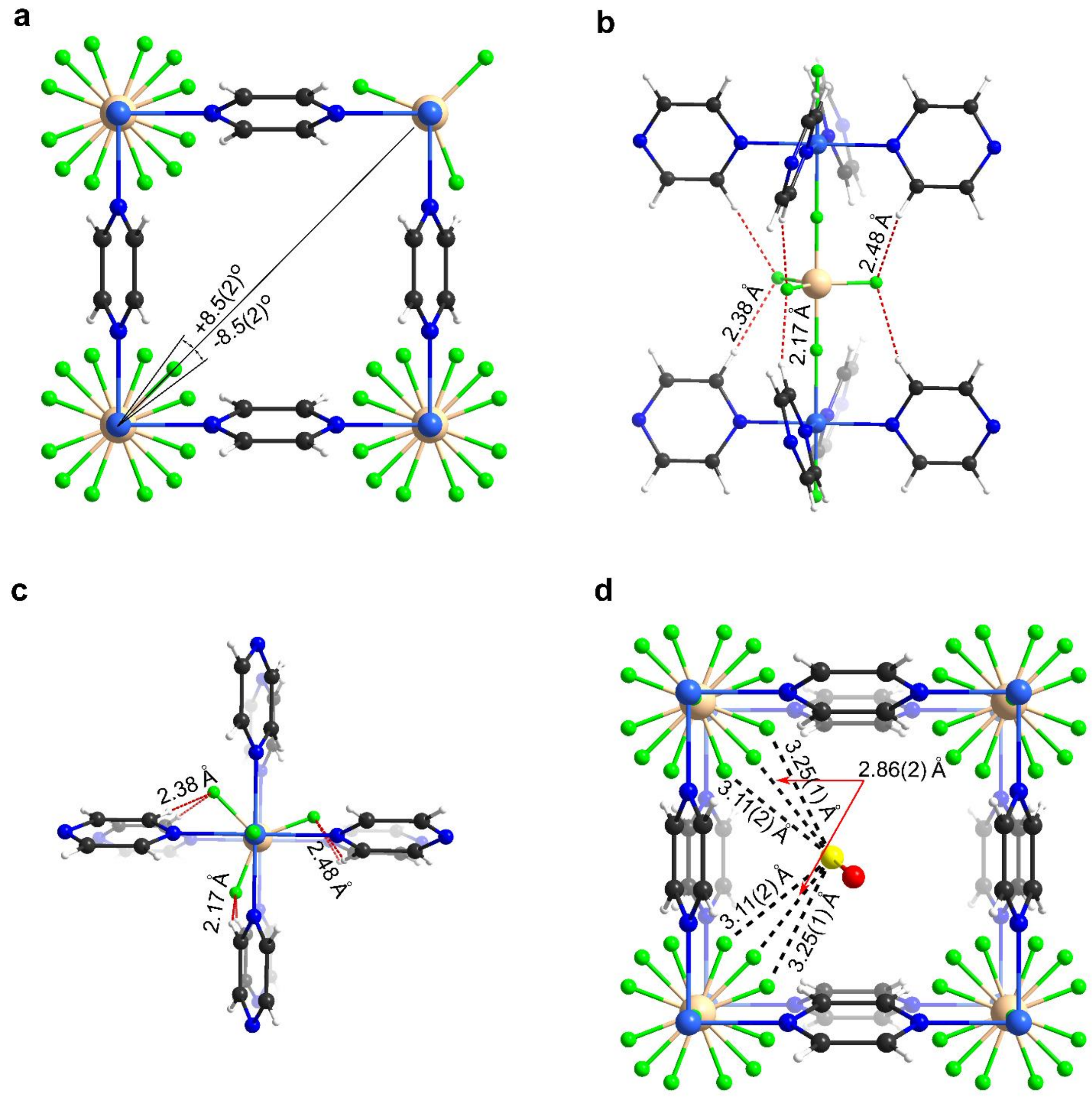

Supplementary Figure 9. KAUST-8 (SO_2) crystal structure: a) $(AlF_5)^{2-}$ disorder; Pyrazine-pillar C–H···F hydrogen bonding side b) and top c) view. Only one orientation of $(AlF_5)^{2-}$ is shown; d) $(AlF_5)^{2-}$-SO_2 interactions: distances F···S depends on the pillar orientation.

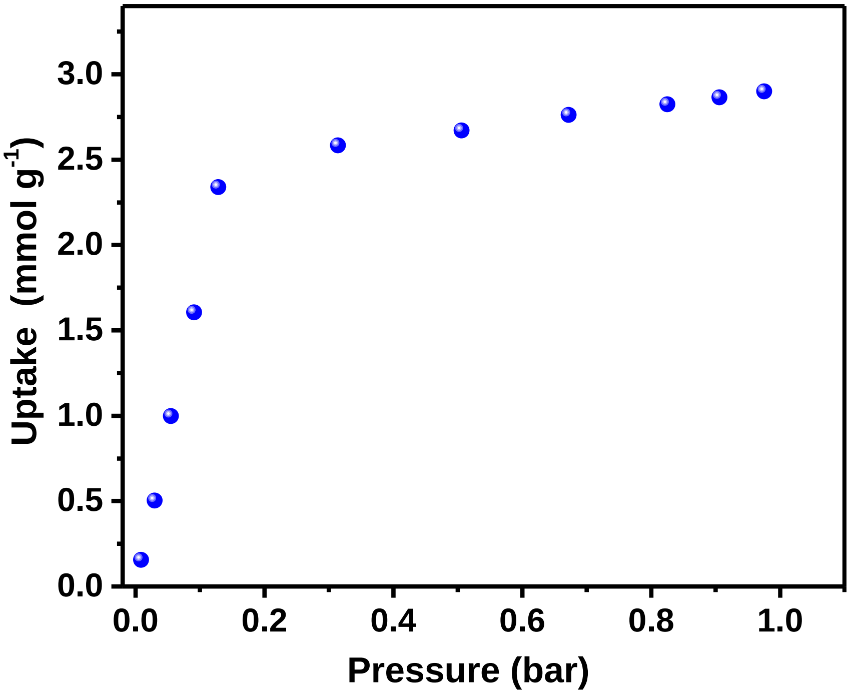

Supplementary Figure 10. SO_2 isotherm for KAUST-8 at 25 °C after 105 °C activation

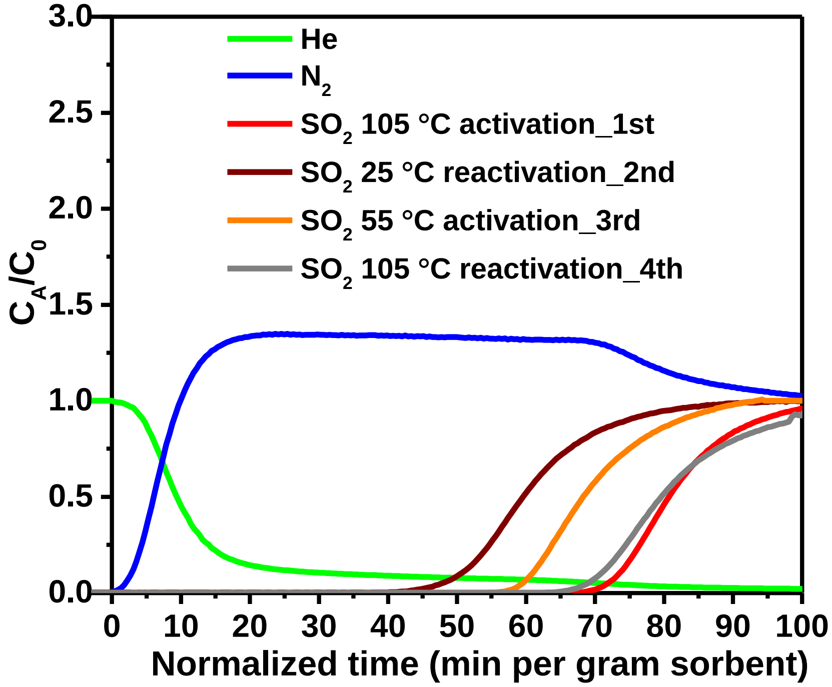

Supplementary Figure 11. Cyclic breakthrough experiments for KAUST-8 using 7% SO_2 (balance N_2) gas mixture feed (10cc/min) for optimisation of regeneration temperature. The experiments also confirm stability and recyclability of the material.

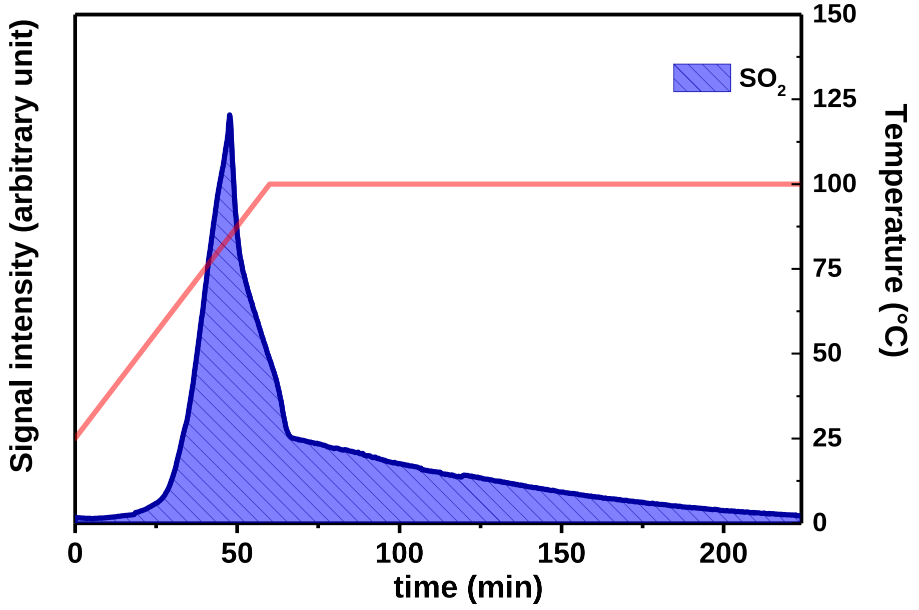

Supplementary Figure 12. TPD analysis of adsorbed phase for KAUST-8 after breakthrough experiments with 500 ppm SO_2 (balance N_2).

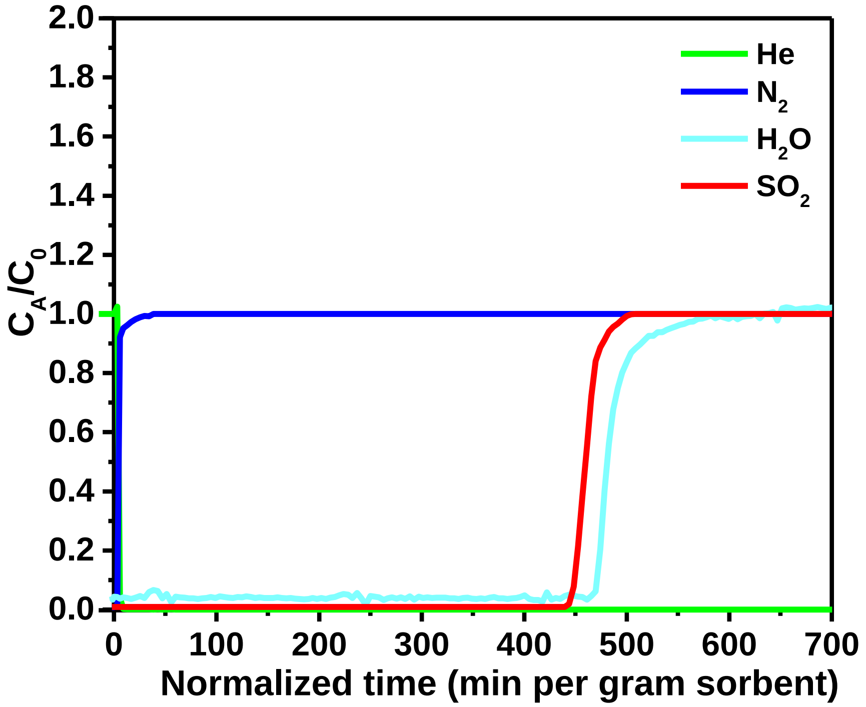

Supplementary Figure 13. Breakthrough experiment for KAUST-8 using humid (≈40% RH) 250 ppm SO_2/ balance N_2 gas mixture feed at the flow rate of 50cc/min (1bar, 298K).

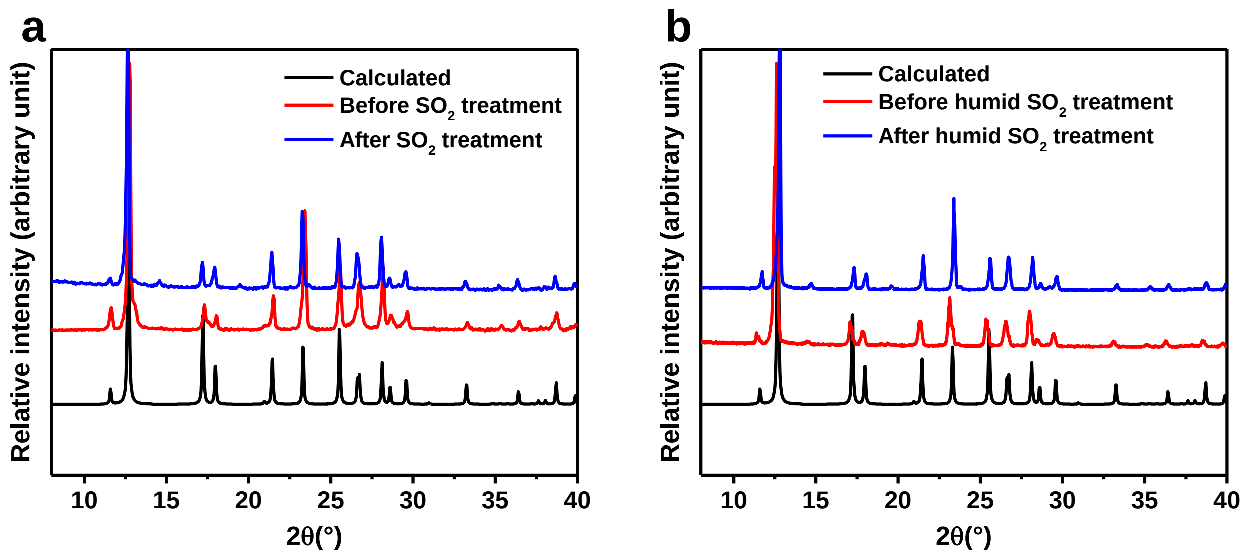

Supplementary Figure 14. (a) Comparison of PXRD patterns of KAUST-8 before (red) and after SO_2 exposure (blue) with calculated PXRD (black) from the single crystal structure. **(b)** Comparison of PXRD patterns of KAUST-8 before (red) and after humid SO_2 exposure (blue) with calculated PXRD (black) from the single crystal structure.

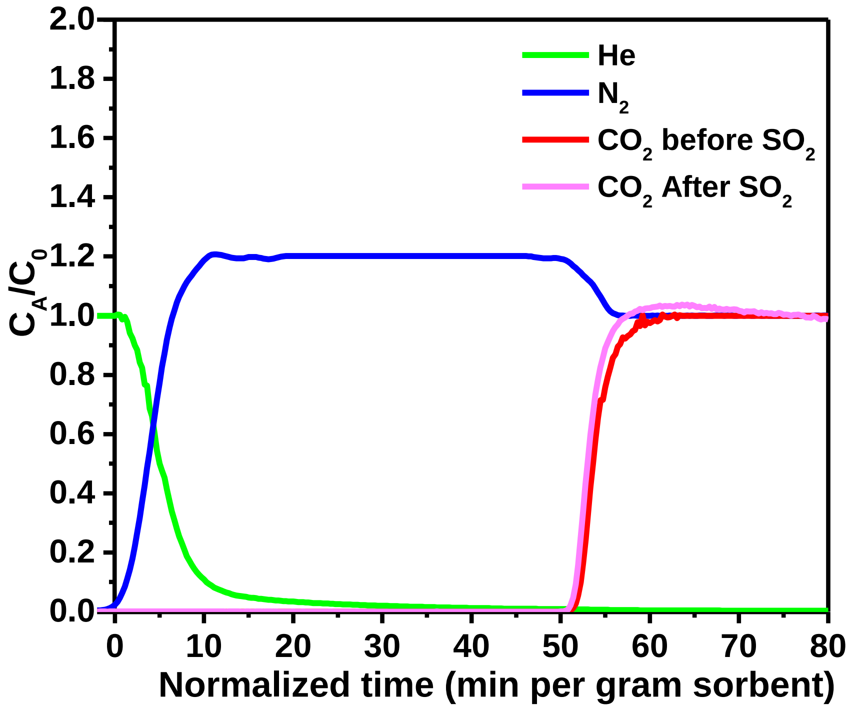

Supplementary Figure 15. Breakthrough experiments for KAUST-8 using 10% CO_2/balance nitrogen feed (10 cc/min) before and after 7% SO_2 (balance N_2) breakthrough experiments.

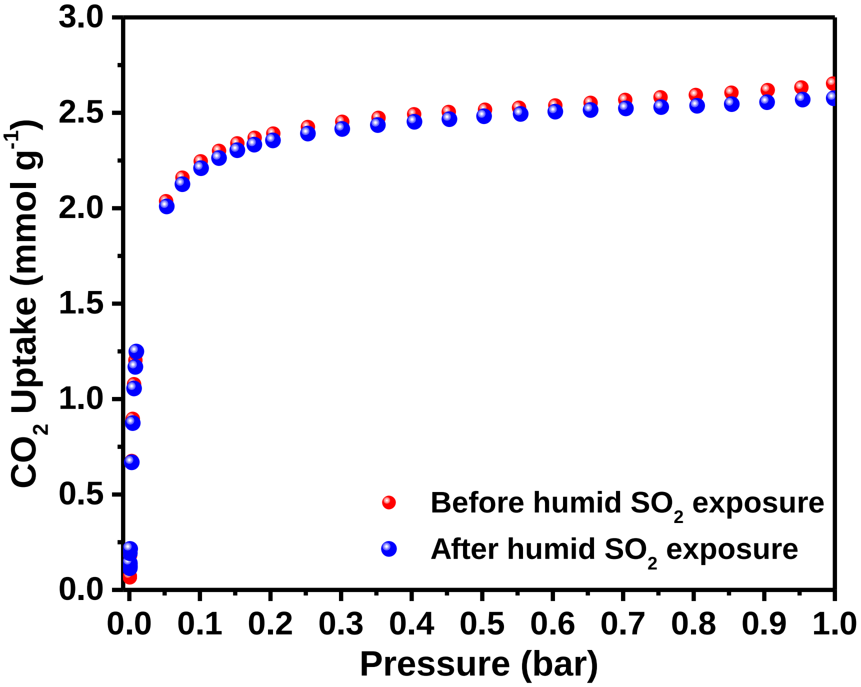

Supplementary Figure 16. CO_2 isotherms at 298 K for KAUST-8 before and after breakthrough experiment with humid (≈40% RH) 250 ppm SO_2/ balance N_2 gas mixture.

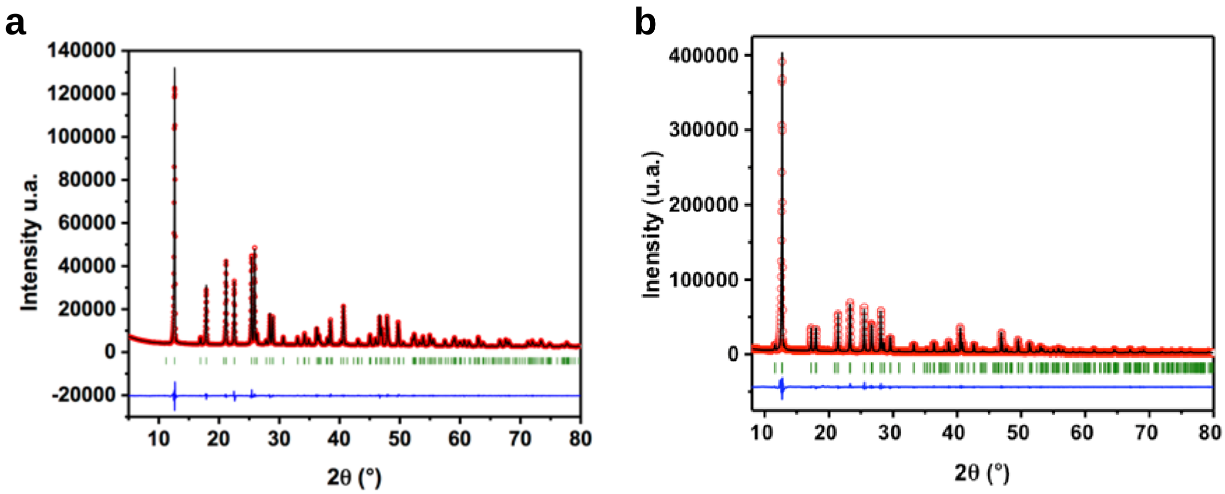

Supplementary Figure 17. Final Le Bail profile refinement with observed (black line), calculated (red point), and difference (blue line) profiles of X-ray of diffraction data, vertical green bars are related to the calculated Bragg reflection positions. (a) KAUST-7 (Rp = 0.074, Rwp = 0.079, Rexp = 0.031, $\chi 2$ = 6.73). (b) KAUST-8 (Rp = 0.086, Rwp = 0.108, Rexp = 0.022, $\chi 2$ = 23.1)

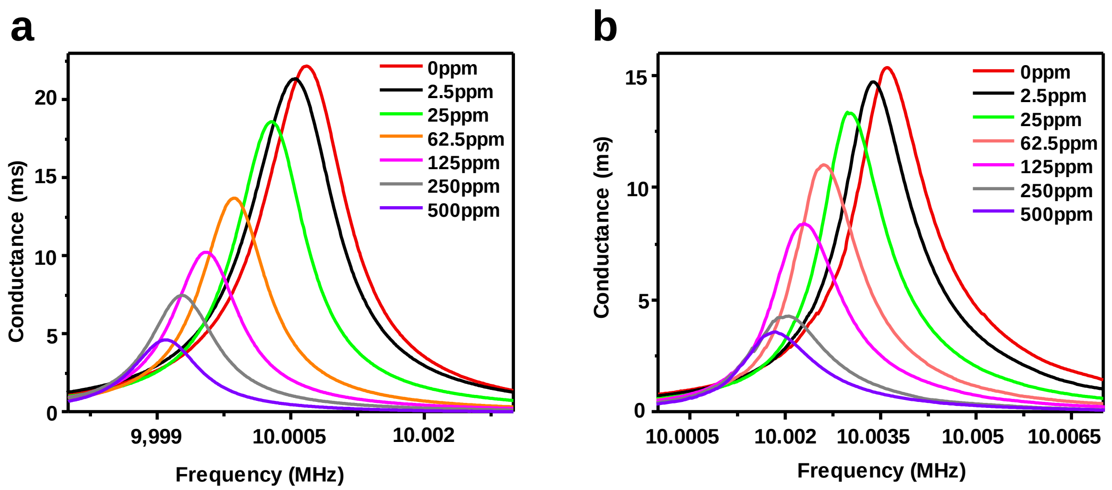

Supplementary Figure 18. Variation of the peak resonance frequency (a) KAUST-7 (b) KAUST-8, in response to the introduction of various concentrations of dry SO_2.

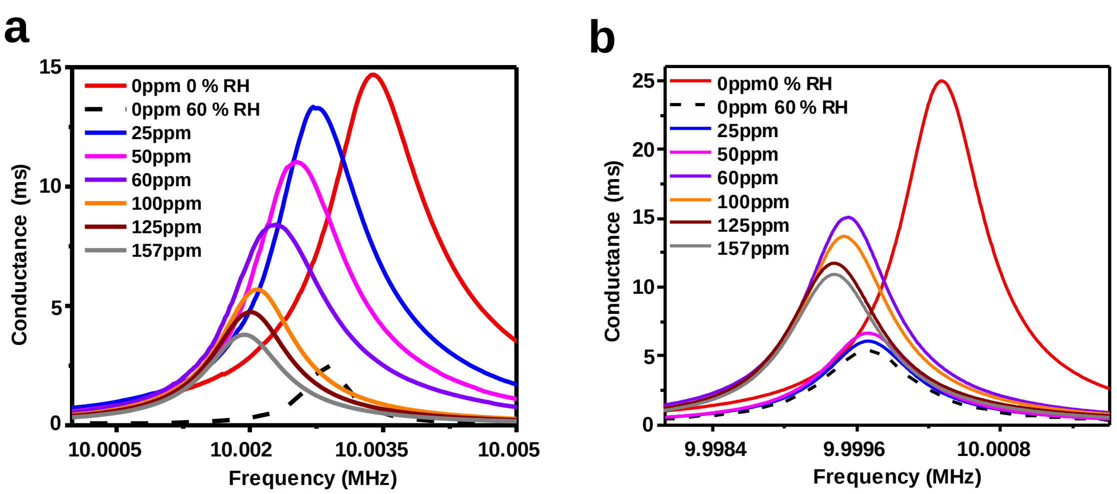

Supplementary Figure 19. Variation of the peak resonance frequency (a) KAUST-7 (b) KAUST-8, in response to the introduction of various concentrations of humid SO_2.

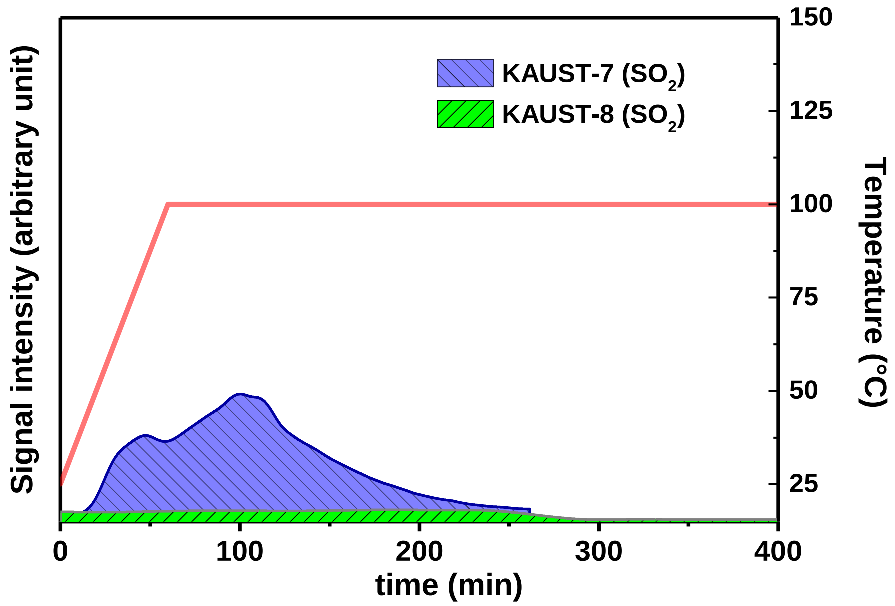

Supplementary Figure 20. Temperature-programed desorption (TPD) of SO_2. During typical experiment, materials were saturated with water at 60% RH by flowing humid He. After water saturation (as detected by mass spectrometer), humid He flow was allowed to continue for two more hours. At this point, gas flow was changed to 500 ppm SO_2 with balance N_2 (dry, 23 cc/min flow rate) for two hours. Adsorbed phase was analysed by TPD experiment by increasing the temperature of the column under He flow (15 cc/min). The TPD experiment results show that in the case of KAUST-7, SO_2 is able to replace adsorbed water relatively easily than KAUST-8. The results are on expected line considering the relative water affinity of both the compound and further support the trend in sensing experiments.

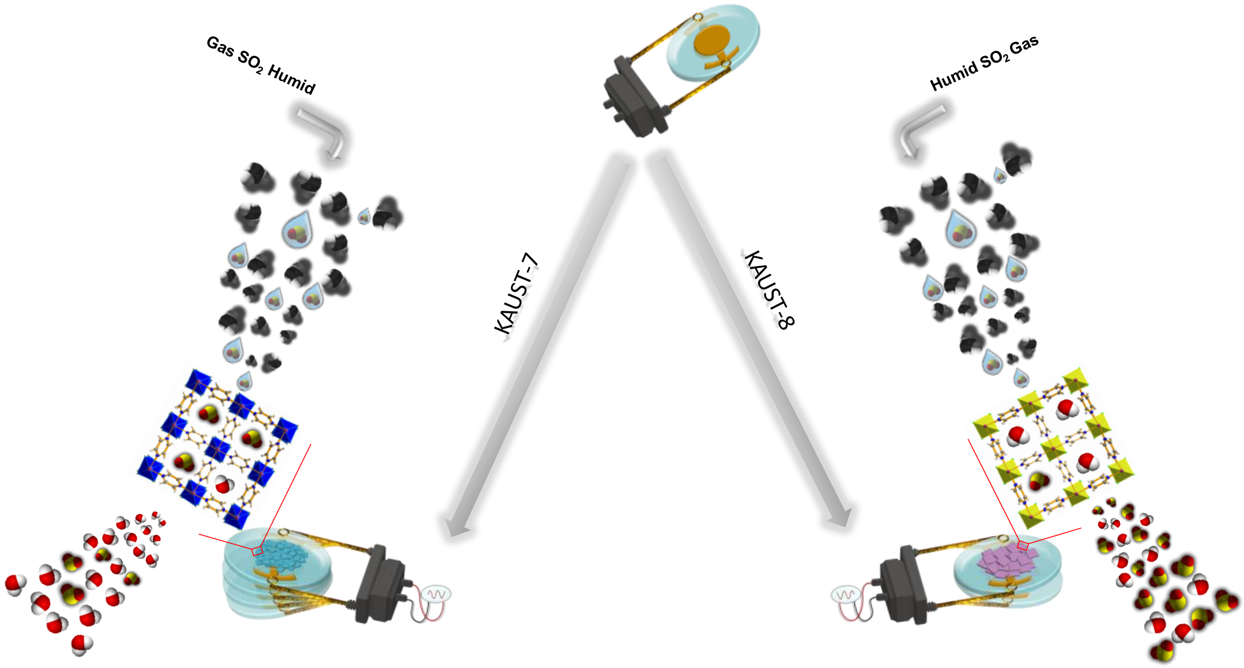

Supplementary Figure 21. Illustration of the selective removal and sensing of SO_2 from air using fluorinated MOF Platform KAUST-7 and KAUST-8.

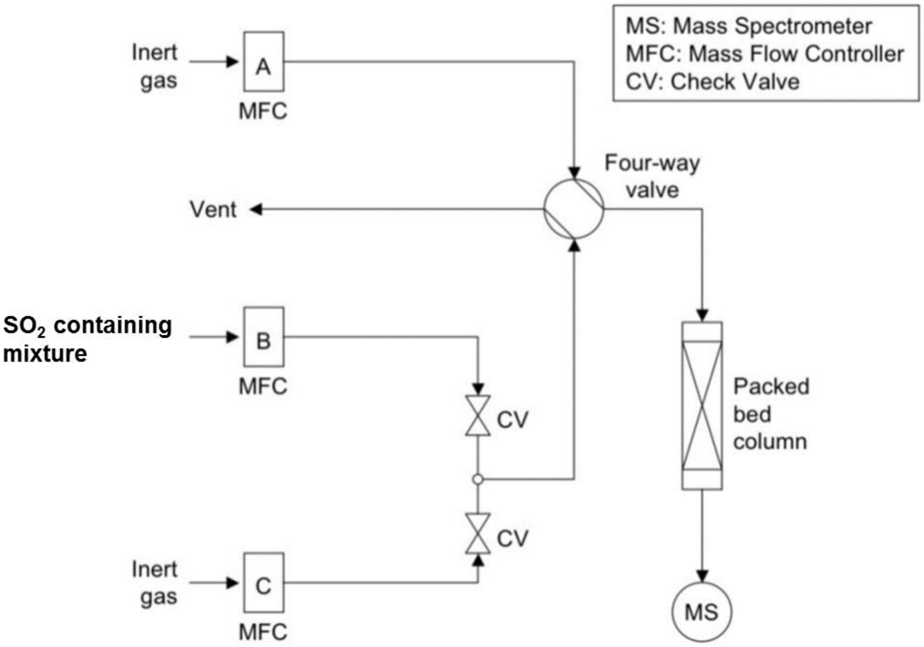

Supplementary Figure 22. Schematic representation of the column breakthrough set-up.

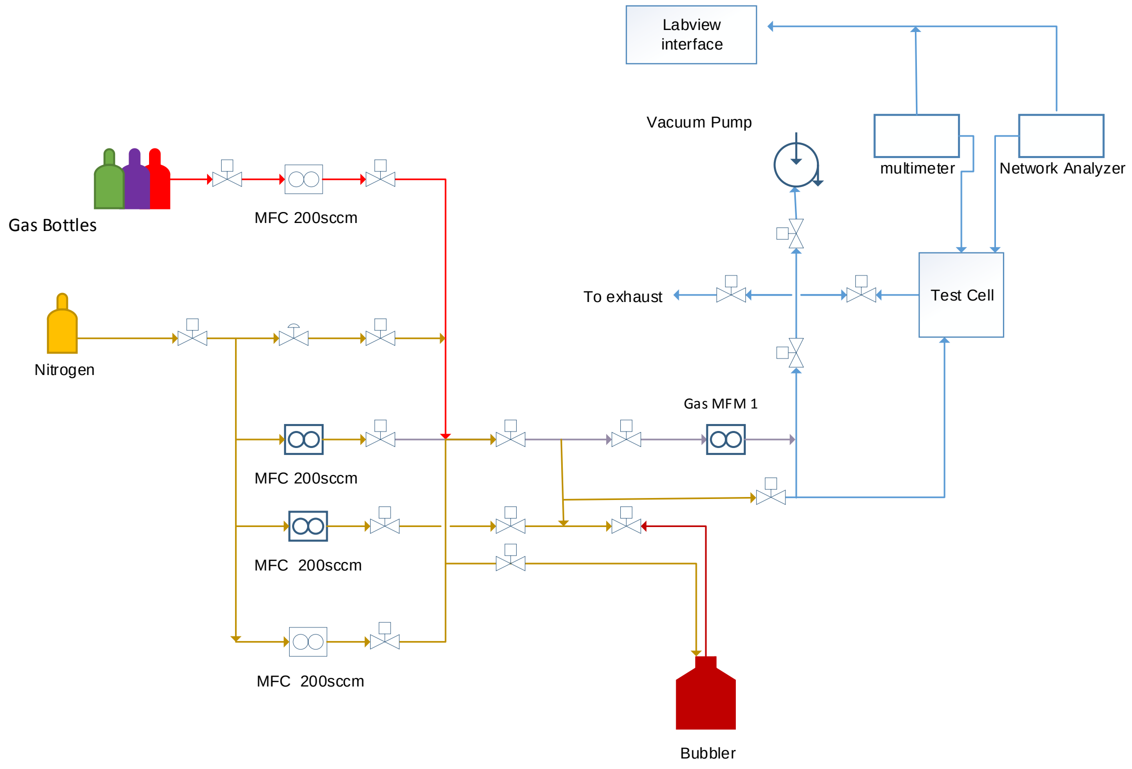

Supplementary Figure 23. Illustrative scheme of gas flow control and dilution system and sensor measurement set-up.

Supplementary Tables

Supplementary Table 1. Crystal data and structure refinement conditions for KAUST-7 (SO_2)

Empirical formula	$C_8H_8F_5N_4NbNiO_{1.84}S_{0.42}$
Formula weight	449.79
Crystal system, space group	Tetragonal, *P*4/*nbm*
Unit cell dimensions	a = 9.9249(2) Å, c = 7.8387(2) Å
Volume	772.14(4) Å^3
Z, calculated density	2, 1.935 Mg m^{-3}
F(000)	439
Temperature (K)	296(2)
Radiation type, λ	Cu $K\alpha$, 1.54178 Å
Absorption coefficient	8.71 mm^{-1}
Absorption correction	Multi-scan
Max and min transmission	0.149 and 0.034
Crystal size	0.01 × 0.01 × 0.02 mm
Shape, colour	Square prism, light blue
θ range for data collection	6.3–66.5°
Limiting indices	$-11 \le h \le 11$, $-11 \le k \le 11$, $-9 \le l \le 9$
Reflection collected / unique / observed with $I > 2\sigma(I)$	5721 / 374 (R_{int} = 0.061) / 300
Completeness to θ_{max} = 65.7°	99.7 %
Refinement method	Full-matrix least-squares on F^2
Data / restraints / parameters	374 / 10 / 39
Final R indices [$I > 2\sigma(I)$]	R_1 = 0.039, wR_2 = 0.102
Final R indices (all data)	R_1 = 0.046, wR_2 = 0.107
Weighting scheme	$[\sigma^2(F_o^2) + (0.0620P)^2 + 0.4065P]^{-1*}$
Goodness-of-fit	1.14
Largest diff. peak and hole	0.72 and -0.22 e Å^{-3}

$^*P = (F_o^2 + 2F_c^2)/3$

Supplementary Table 2. Crystal data and structure refinement conditions for KAUST-8 (SO_2)

Empirical formula	$C_8H_8AlF_5N_4NiO_{1.91}S_{0.95}$
Formula weight	401.89
Crystal system, space group	Tetragonal, *P*4/*mmm*
Unit cell dimensions	a = 6.9996(2) Å, c = 7.7033(2) Å
Volume	377.42(2) Å^3
Z, calculated density	1, 1.768 Mg m^{-3}
F(000)	200
Temperature (K)	296(2)
Radiation type, λ	Cu $K\alpha$, 1.54178 Å
Absorption coefficient	4.29 mm^{-1}
Absorption correction	Multi-scan
Max and min transmission	0.147 and 0.042
Crystal size	0.01 × 0.02 × 0.02 mm
Shape, colour	Square prism, blue-violet
θ range for data collection	5.7–65.5°
Limiting indices	$-7 \leq h \leq 7, -8 \leq k \leq 7, -7 \leq l \leq 9$
Reflection collected / unique / observed with $I > 2\sigma(I)$	2108 / 231 (R_{int} = 0.023) / 222
Completeness to θ_{max} = 65.4°	99.6 %
Refinement method	Full-matrix least-squares on F^2
Data / restraints / parameters	231 / 13 / 37
Final R indices [$I > 2\sigma(I)$]	R_1 = 0.034, wR_2 = 0.097
Final R indices (all data)	R_1 = 0.035, wR_2 = 0.098
Weighting scheme	$[\sigma^2(F_o^2) + (0.0731P)^2 + 0.1226P]^{-1*}$
Goodness-of-fit	1.13
Largest diff. peak and hole	0.43 and -0.15 e Å^{-3}

$^*P = (F_o^2 + 2F_c^2)/3$

Supplementary Table 3. A comparison of the SO_2 uptake properties of the reported materials compared with those of the MOF in the present study at ppm level

**Material**	**SO_2 uptake (mmol/g)**	**SO_2 concentration**	**Reference**	**Remarks**
**KAUST-7**	1.4 ^	500 ppm	This work	Proven stability to SO_2 and moisture
**KAUST-8**,	1.6^	500 ppm	This work	Proven stability to SO_2 and moisture
**SIFSIX**-1-Cu	1.8^	2000 ppm	Ref 1	Not stable to moisture
**SIFSIX**-2-Cu-i	2.31 ^	2000 ppm	Ref 1	Not stable to moisture
**SIFSIX**-3-Zn	0.98^	2000 ppm	Ref 1	Not stable to moisture
**SIFSIX**-3-Ni	1.39^	2000 ppm	Ref 1	Not stable to moisture
**MFM-300(In)**	≈1§	1%	Ref 2	Proven stability to SO_2 and moisture
**NOTT-300 (Al)**	0.9-1ǂ	5000 ppm	Ref 3	Proven stability to SO_2 and moisture

^ Calculated from mixed gas column breakthrough experiments under dynamic condition
§ Estimated from SO_2 isotherm available in ESI of reference 2.
ǂ Derived from SO_2 isotherm data retrieved from: Siderius, D.W., Shen, V.K., Johnson III, R.D. and van Zee, R.D., Eds., NIST/ARPA-E Database of Novel and Emerging Adsorbent Materials, National Institute of Standards and Technology, Gaithersburg MD, 20899, https://dx.doi.org/10.18434/T43882.

Supplementary Note 1
Single-crystal X-ray diffraction data

Crystals of both materials, KAUST-7 (SO_2) and KAUST-8 (SO_2) MOFs, are isostructural. Crystal data and refinement conditions are shown in Supplementary Table 1 and Supplementary Table 2 for KAUST-7 (SO_2) and KAUST-8 (SO_2), respectively. Disorder of a pyrazine ring (50:50) in KAUST-8 (SO_2) changes the symmetry and results in the space group *P*4/*mmm* with $a_{Al} \approx 2^{-½}a_{Nb}$ and $c_{Al} \approx c_{Nb}$ compared to KAUST-7 (SO_2) crystallizes in the space group *P*4/*nbm* (Supplementary Table 1 and Supplementary Table 2).

In the case of KAUST-7 (SO_2) structure, pseudo-octahedral $[NbOF_5]^{2-}$ anion is deformed due to different axial ligands (O^{2-} or F^-) and, therefore, different Nb–O / Nb–F bond lengths. The Nb was splitted into two positions. Atoms F1 and O1 were refined at the same position with the equal thermal parameters (EXYZ and EADP). Thermal parameters of F2 atom of disordered $[NbOF_5]^{2-}$, were restrained by ISOR 0.02. The guest SO_2 molecule was refined isotropically to increase data-to-parameter ratio. The S=O bonds were restrained by DFIX 1.431 0.01 and 1,3- O···O distances were restrained by DFIX 2.46. The PLATON's SQUEEZE procedure was used to estimate an electron density within the KAUST-7 pores. Localized 12.8 ē/pore corresponds to 12.8/(16 + 2·8) = 12.8/32 = 0.4 SO_2 molecule per pore and it is close to the refined value of 0.424(4).

The trigonal bypiramidal $[AlF_5]^{2-}$ anion in KAUST-8 (SO_2) is disordered due to rotation around axial Al–F bonds over four orientations with an equal occupancy (0.25). Thermal parameters of F2 and F3 atoms of disordered $[AlF_5]^{2-}$ were restrained by ISOR 0.02 and ISOR 0.01, respectively. The guest SO_2 molecule was refined isotropically to increase data-to-parameter ratio. The S=O bond was restrained by DFIX 1.431 0.01. The electron density estimation (SQUEEZE) within the KAUST-8 pores results in 31.1 ē/pore, which corresponds to 31.1/(16 + 2·8) = 31.1/32 = 0.972 SO_2 molecule per pore and it is close to the refined value of 0.956(5).

In both KAUST-7 (SO_2) and KAUST-8 (SO_2) crystal structures, hydrogen atoms are placed at calculated positions and refined using a riding model with $U_{iso}(H) = 1.2U_{eq}(C)$.

References:

1. Cui, X. *et al.* Ultrahigh and Selective SO_2 Uptake in Inorganic Anion-Pillared Hybrid Porous Materials. *Adv. Mater.* **29** (2017).
2. Mathew, S. *et al.* Selective Adsorption of Sulfur Dioxide in a Robust Metal–Organic Framework Material. *Adv. Mater.* **28**, 8705 (2016).
3. Yang, S. *et al.* Selectivity and direct visualization of carbon dioxide and sulfur dioxide in a decorated porous host. *Nat. Chem.* **4**, 887 (2012).
